# Validation of a low-cost continuous renal replacement therapy dialysate fluid controller for experimental purposes

**DOI:** 10.1186/s40635-024-00593-z

**Published:** 2024-02-02

**Authors:** Yuri de Albuquerque Pessoa dos Santos, Viviane Flor Park, Luis Carlos Maia Cardozo Junior, Bruno Adler Maccagnan Pinheiro Besen, Pedro Vitale Mendes, Marcelo Park

**Affiliations:** 1 Intensive Care Unit, Hospital das Clínicas, University of São Paulo Medical School, São Paulo, SP Brazil; 2 Laboratory of Medical Investigation (LIM-51), Emergency Discipline, University of São Paulo Medical School, São Paulo, SP Brazil; 3 Institute of Science and Technology, Federal University of São Paulo, São José dos Campos, SP Brazil

**Keywords:** Acute kidney injury, Continuous renal replacement therapy, Intensive care units, Experimental dialysis, Cost analysis

## Abstract

**Background:**

Continuous renal replacement therapy (CRRT) support is crucial for critically ill patients and it is underexplored in specific situations. Experimental CRRT offers a means to gain insights into these scenarios, but the prohibitive cost of CRRT machines limits their accessibility. This study aimed to develop and validate a low-cost and precise dialysate controller for experimental CRRT.

**Results:**

Our results demonstrate a commendable level of precision in affluent flow control, with a robust correlation (*R*^2^ = 0.99) for continuous flow and a strong correlation (*R*^2^ = 0.95) for intermittent flow. Additionally, we observed acceptable agreement with a bias = 3.4 mL (upper limit 95% = 43.9 mL and lower limit 95% = − 37 mL) for continuous flow and bias = − 20.9 mL (upper limit 95% = 54 mL and lower limit 95% = − 95.7 mL) for intermittent flow, in this way, offering a precise CRRT dose for the subjects. Furthermore, we achieved excellent precision in the cumulative ultrafiltration net (UFnet), with a bias = − 2.8 mL (upper limit 95% = 6.5 mL and lower limit 95% = − 12 mL). These results remained consistent even at low affluent flow rates of 8, 12, and 20 mL/min, which are compatible with CRRT doses of 25–30 mL/kg for medium-sized animals. Moreover, the acceptable precision of our findings persisted when the dialysate controller was subjected to high filter dialysate chamber pressure for an extended duration, up to 797 min.

**Conclusions:**

The low-cost dialysate controller developed and tested in this study offers a precise means of regulating CRRT in experimental settings. Its affordability and accuracy render it a valuable instrument for studying CRRT support in unconventional clinical scenarios, particularly in middle-income countries’ experimental ICU laboratories.

**Supplementary Information:**

The online version contains supplementary material available at 10.1186/s40635-024-00593-z.

## Background

Continuous renal replacement therapy (CRRT) is a crucial support for critically ill patients with severe acute kidney injury (AKI). In high-income countries, CRRT is used in 80% of intensive care unit (ICU) patients who need renal replacement therapy (RRT); although, these CRRT-supported patients represent only 3.8% of patients admitted to the ICU [1]. The literature about CRRT use in specific, less frequent, clinical scenarios potentially associated with AKI, such as exogenous intoxications, severe metabolic acidosis, and severe respiratory acidosis, is scarce.

Currently, large animal models accurately and faithfully replicate severe pathophysiological states and the critical care interventions entailed therein, encompassing many syndromes [2–12], as well as CRRT use [6]. Experimental CRRT brings us the possibility to study renal support in those unusual clinical scenarios [6]. However, the operational costs associated with maintaining a CRRT apparatus are substantial [13, 14]. The high demand for CRRT machines within ICU settings also precludes their exclusive allocation for the purpose of experimental research [15]. Hence, the development of a cost-effective and accurate CRRT machine purposefully designed for experimental investigations emerges as an intriguing prospect, particularly within the context of experimental ICU laboratories in middle-income countries [3].

This study aimed to conceive, construct, and validate a low-cost and accurate dialysate controller for CRRT in experimental models of critical care syndromes.

## Methods

The bench experiments were conducted at the Laboratory of Medical Investigation 51 (LIM-51), affiliated with the University of São Paulo Medical School. Given the nature of these experiments, which exclusively involve the principles of the dialysate controller, device construction, and a sequence of bench experiments, and did not involve the use of animals, there was no requirement for evaluation or approval from the Experimental Research Ethics Committee.

### Dialysate controller principles

The dialysate controller offers the affluent fluid for diffusion to the metabolic filter and/or fluids for patient reposition when convective clearance is used. The difference between the effluent and afferent fluid flows is the net ultrafiltration (UFnet), which allows for extracorporeal fluid management in patients/subjects. The dialysate controller must be precise in offering the set affluent volume and controlling the effluent fluid, therefore generating a precise UFnet. Along the time the affluent and effluent flow must be homogeneously distributed, as well as the UFnet.

### Building the dialysate controller

We built the dialysate controller using two peristaltic pumps to control the affluent and effluent flows. Peristaltic pumps offer a continuous flow according to the number of rotations of the impeller per minute. Additionally, the number of rotations is linearly correlated to the energy power supplied [16]. The peristaltic pumps are free from preload and afterload modulation. The peristaltic pumps fluid flow has a ceiling effect determined by the pipes inside the pump resistance, that is, the lesser the inside pump conduit diameter, the higher the resistance for fluid flow, and, at last, the lower is the highest flow reachable. The resistance limits the maximal fluid flow due to inlet pump negative pressure, perhaps ending in circuit collapse, as well as the outlet pump fluid flow limitation [17]. In this way, a backup mechanism is necessary to adjust the flows (affluent and effluent) warranting a stable and preset UFnet. We have used two strain gauges (balances) as a backup servo-controlled mechanism, controlling the affluent and effluent bag weight through time, allowing the fluid flow and UFnet control.

We have used a third peristaltic pump to infuse heparin in the dialysis filter blood inlet. This pump was preset to offer one milliliter of the injected solution every 10 min. All backup components, the three peristaltic pumps, and the two balances were servo-controlled using an Arduino’s ™ microcontroller [18]. Additional file 1: Figure S1 shows the schematic view, the final design, and the components of the controller.

### Power and strain gauge amplifiers

The Arduino’s microcontroller power source was supplied by the microcomputer (Toshiba™ Satellite—L635) USB port (5 V and 500 mA). In order to supply the three motors with enough energy power at the same time, three conventional power amplifiers, based on Darlington’s transistors, were used. An independent electrical power source of 13 V and 750 mA is supplied directly to the peristaltic pump motors; the single electronic circuit is shown in Additional file 1: Fig. S2.

In order to amplify the strain gauges (balances) output signal to the Arduino’s microcontroller analogic input port, a load amplifier HX711 was used, the electronic and electrical circuits are shown in Additional file 1: Fig. S3.

### Arduino’s principles

Arduino is an open-source programmable and easy-to-use circuit board. With the guidance of an integrated development and learning environment (IDLE)—written sketch, it receives several inputs that are processed by the microcontroller at its core, producing corresponding outputs [19]. The dialysis machine was built using an Arduino UNO R3. It has an input/output voltage of 5 V, an ATmega328P 16 MHz main processor, 14 digital input/output pins and 6 analogic input pins [20].

### Measurement techniques

The volume of fluids was measured using a common beaker (shown in Additional file 1: Figs. S1 and S4). The weight of the fluid was considered equal to the volume (density = 1); moreover, the weight variation is the same as the volume variation. We obtained the flow measure integrating weight and/or volume variation for a given period of time.

The beaker was suspended in the strain gauge (Additional file 1: Fig. S1—panel C) to measure weight and volume variation through time. The instantaneous fluid bag weight, time, and bits output from a microcontroller to the pump motors were read through the serial monitor of the Arduino’s microcontroller IDLE of the microcomputer. The voltages offered to the pump motors were measured using an analogic multimeterHioki™ 1001 (Nagano, Japan).

### Materials and costs

The materials used to build the dialysate controller are listed in Additional file 1: Table S1 with their respective costs. The total cost of the device was 53.87 (United States Dollars) USD.

### Validation sequence

Three different configurations were used to collect data for validation of the machine. Additional file 1: Figure S4 shows them. Normal saline 0.9% was used as the test fluid.

Using configuration 1 we proceeded as follows:

Experiment 1: Values of bits output from the microcontroller were arbitrarily chosen from 10 to 120 and set through the IDLE. The values chosen were:

- Pump 1–25, 30, 35, 40,50,60,70, 75, 80, 90 and 120.
- Pump 2–10, 20, 23, 25,30, 40, 42, 45, 50, 60, 70, 80, 90, 100, 110 and 120.

The pump flow with each value of bits output was executed for a period of 1 min. At the end of this observation period, the difference in potential of the evaluated pump (in Volts), the resultant fluid volume (measured with the beaker), and flow (the volume measured integrated with the time) was then collected for each step (bits set).

Experiment 2: A fixed volume (500–1000 mL) of fluid was delivered from affluent to the effluent balance using a given number of bits output from the microcontroller. Pump 2 was arbitrarily chosen for this step since both pumps had a similar behavior according to the bits output in experiment 1. The numbers of bits chosen for this step were: 25, 30, 35, 40, 60, 75, 80, 90, and 120.

As the motor of the pumps sometimes stuck when the number of bits output was lower than 25 (probably due to the high friction), we tested the flow with intermittent functioning of the motor pumps. With the intermittent function, when the motor was working, the number of bits output was higher, avoiding the motor stalling. The motors’ period for intermittent service was 4 s on and 4 s off. The number of bits output tested with intermittent work were 5 and 10 bits.

In experiment number 2, we collected the weight of fluid transferred from affluent to the effluent balance (with time points of around 2 s), the volume measured through the beaker (with time points of 2 min), and the resultant flow. These data were also used to measure the correlation and agreement of the weight and beaker volume of fluid, as well as the fluid flow obtained from weight and beaker volume variation during the time. All data were collected with continuous and intermittent flow.

Experiment 3: During this experiment a transfer of 500 mL of normal saline from the afferent balance to the efferent balance, using pump 1, with a preset flow of 8, 12, and 20 mL/min was tested. To keep the pre-adjusted flow, the sketch (see below) loaded to the microcontroller, based on the algorithm of Additional file 1: Fig. S5, was used. The number of bits output was variable according to the algorithm. To maintain the flow as stable as possible, some periods with the motor pumps off were allowed. This experiment tested the capacity of the dialysate controller to keep a stable preset fluid flow dynamically adjusting the number of bits output from the microcontroller according to the affluent weight variation. The flows of 8, 12, and 20 mL/min were chosen to reproduce a continuous renal replacement therapy dose of 20 to 40 mL/kg/h in animals of around 30 kg of weight.

Using configuration 2 we proceeded as follows:

Experiment 4: During this experiment, we have used both sets of pumps and balances. The beaker was used as a fluid (dialysate) compartment of the dialysis filter (a surrogate of patient/subject UFnet). The affluent and effluent lines were attached to the beaker filled with 100 mL of normal saline. The UFnet values tested were 0, 50, and 100 mL/h. Each subset of the experiment lasted 2 h.

In this experiment, we measured the affluent and effluent volume, the affluent flow through the time, the cumulative UFnet, and at last the correlation and agreement of the cumulative UFnet measured through the fluid bags weight variation and predicted UFnet. The predicted UFnet was calculated by dividing UFnet (per hour) for 60 min and cumulative addition of this value to each minute observed during the experiment.

Using configuration 3 we proceeded as follows:

Experiment 5: In this experiment, the beaker volume was not collected. The fluid bag’s weight was used as a beaker volume surrogate. Two experiments lasting 797 and 362 min, respectively, were done. The CRRT dose and UFnet set were 900 mL, 80 mL/h, and 900 mL, 60 mL/h, respectively, for both sets of experiments. The affluent and effluent lines were attached to the normal saline bag filled with 500 mL. This normal saline bag, which was simulating the fluid/dialysate compartment of the dialyzer, was pressurized to 150 mmHg with the cuff (see Additional file 1: Fig. S4) and kept with this pressure throughout the experiment duration.

The weight data were collected with a 2-s interval of time points. We collected the cumulative affluent volume (weight) and cumulative UFnet through the time.

### The final sketch

Additional file 1: Figure S5 shows the modular structure of the sketch. The sketch was written in C language and is shown in “Methods” section of Additional file 1. The periods with the motor pumps on and off were synchronized to avoid large instantaneous variations of UFnet and to reduce or avoid the backfiltration effect, which could potentially reduce the solute clearance [21]. The synchronized affluent and effluent flow was associated with a high performance of low molecular weight solute transfer in a previous dialysis study of our group [6].

### Statistics

The behavior of fluid weight and volume through time are shown as scatter plots for each pump and strain gauge. Additionally, when possible and appropriate, a linear or non-linear regression was fitted and the coefficient of correlation (*r*) and coefficient of determination (*R*^2^) were expressed, as well as the final linear equation [22]. The non-linear fitting was done using the non-linear last square estimation method [23]. The correlation analyzes were done using a linear regression [22], and the agreement was quantified through the Bland–Altman diagram, showing the bias and the upper and lower limits of 95% of agreement [24]. The R free-source software was used to do all graphs and statistical analyses [25].

## Results

Results are shown according to the experiment.

### Experiment 1

**Fig. 1:**
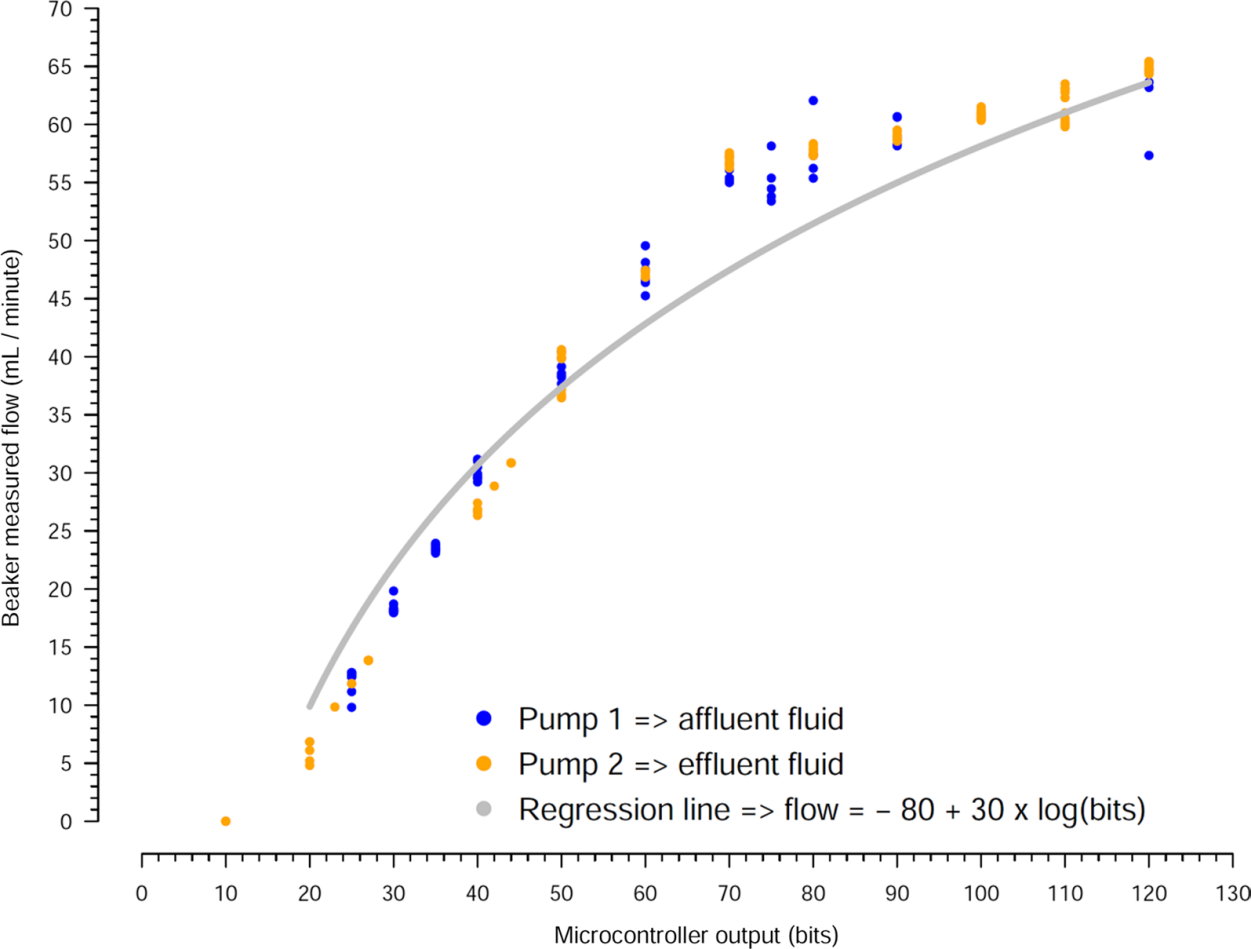
Correlation between the microcontroller output in bits and the resultant fluid flow, measured through the volume quantification using a beaker. The pump 1 (affluent) and pump 2 (effluent) were tested, with 120 and 100 time points, respectively. The configuration 1 of Additional file 1: Fig. S4 was used in this experiment

Figure 1 shows the correlation between the bits output from the microcontroller and beaker-measured flow, which was not linear. Additional file 1: Figure S6 shows the correlation between bits output from the microcontroller and the resultant difference of potential. Additional file 1: Figure S7 shows the correlation between the difference of potential and the beaker measure flow, which was also non-linear.

### Experiment 2

**Fig. 2:**
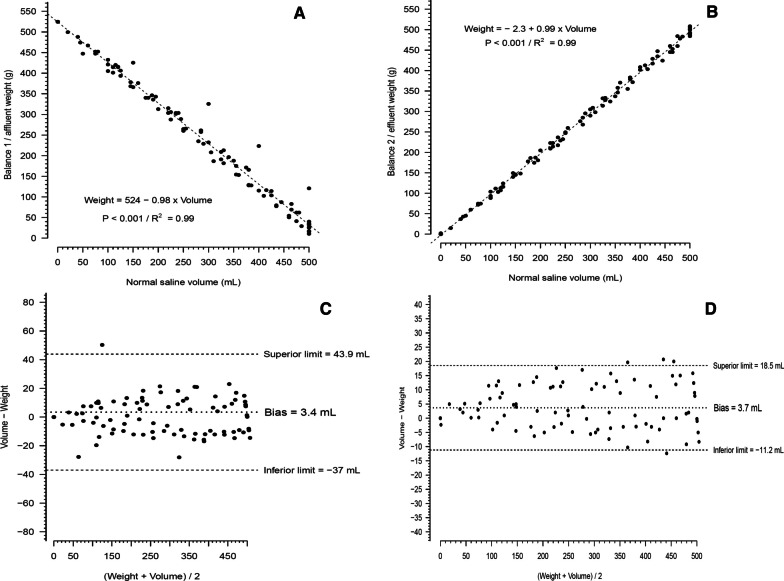
Correlation and agreement between normal saline weight and respective volume measured through the beaker, using the pump 2. **A** Shows the weight decay in balance 1 (affluent) with the real volume transferred to the balance 2 (effluent). **B** Shows normal saline weight gain in balance 2 (effluent) with the volume transferred from the balance 1 (affluent). **C** Shows the Bland–Altman diagram agreement between normal saline weight decay in balance 1 (affluent) with the volume transferred to the balance 2 (effluent). **D** Shows Bland–Altman diagram concordance between normal saline weight gain in balance 2 (effluent) with the real volume transferred from the balance 1 (affluent). The controller configuration 1 of Additional file 1: Fig. S4 was used in this experiment. A total of 101 data points were collected. Pump 2 was arbitrarily chosen since both pumps had a similar behavior during the experiment 1

Additional file 1: Figures S8 to S16 show the behavior of weight and beaker measured volume with the various bits output from the microcontroller over time with the pump motors working continuously. Additional file 1: Figure S17 shows the flow over time with the different bits output tested. Figure 2 shows the excellent correlation and good agreement of the volume measured through the beaker and measured through the weight when the pump motors were working continuously. Additional file 1: Figures S18 and S19 show, respectively, the good correlation and agreement with a relatively low bias of the flow measured either by the weight and by the beaker.

**Fig. 3:**
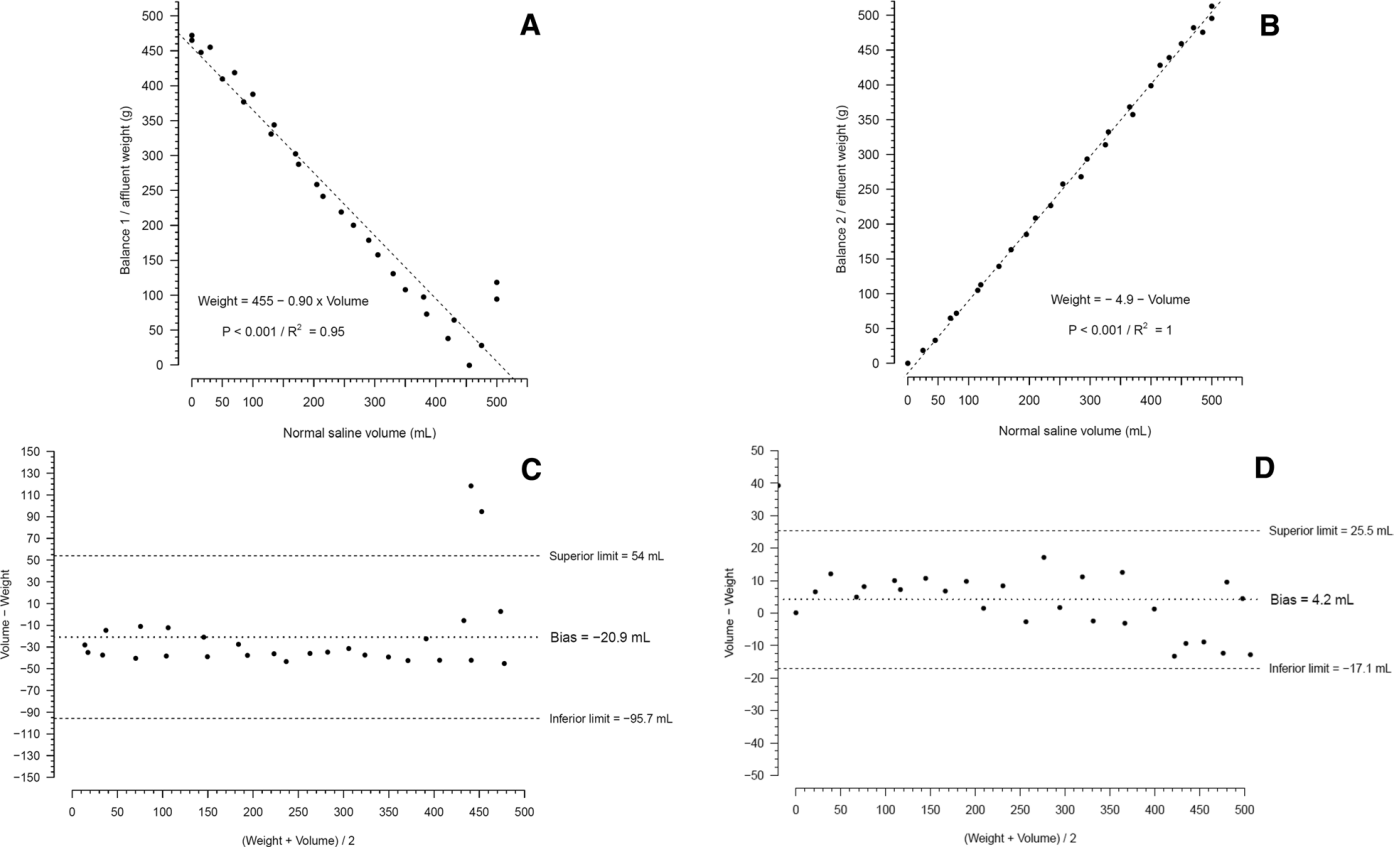
Correlation and agreement of normal saline weight and beaker measured volume, using intermittent flow (approximately 4 s on and 4 s off). The pump number 2 was used. **A** Shows the correlation of the decay of weight and volume in balance 1 (affluent) with the volume transferred to the balance 2 (effluent). **B** Shows the correlation of normal saline weight gain in balance 2 (effluent) with the volume transferred from the balance 1 (effluent). **C** Shows Bland–Altman diagram agreement between normal saline weight decay in balance 1 (affluent) with the volume transferred to the balance 2 (effluent). **D** Shows Bland–Altman diagram agreement between normal saline weight gain in balance 2 (effluent) with the volume transferred from the balance 1 (affluent). The configuration 1 of Additional file 1: Fig. S4 was used in this experiment. Using 5 and 10 bits from microcontroller output. A total of 28 data points were collected. Pump 2 was arbitrarily chosen since both pumps had the same behavior during the experiment number 1

Additional file 1: Figures S20 and S21 show the volume measured through the beaker and weight over time, with intermittent fluid flow. Figure 3 shows the acceptable correlation and agreement of the volume measured through the weight and beaker. Additional file 1: Figure S22 shows the acceptable mean flow over time with the motor pumps working intermittently.

### Experiment 3

Additional file 1: Figure S23 shows the homogeneity of the three different preset flows over the time. Note that the algorithm described in Additional file 1: Fig. S5 and the final sketch (modified to work with just one motor pump) were used to keep the flows constant. The histograms from Additional file 1: Figs. S24–S26 show the occurrences of the number of bits output from the microcontroller using the preset flows of 8, 12, 20 mL/min during this experiment. Additional file 1: Figure S27 shows the linear and homogeneous weight variation of both balances according to the three preset flows.

### Experiment 4

**Fig. 4:**
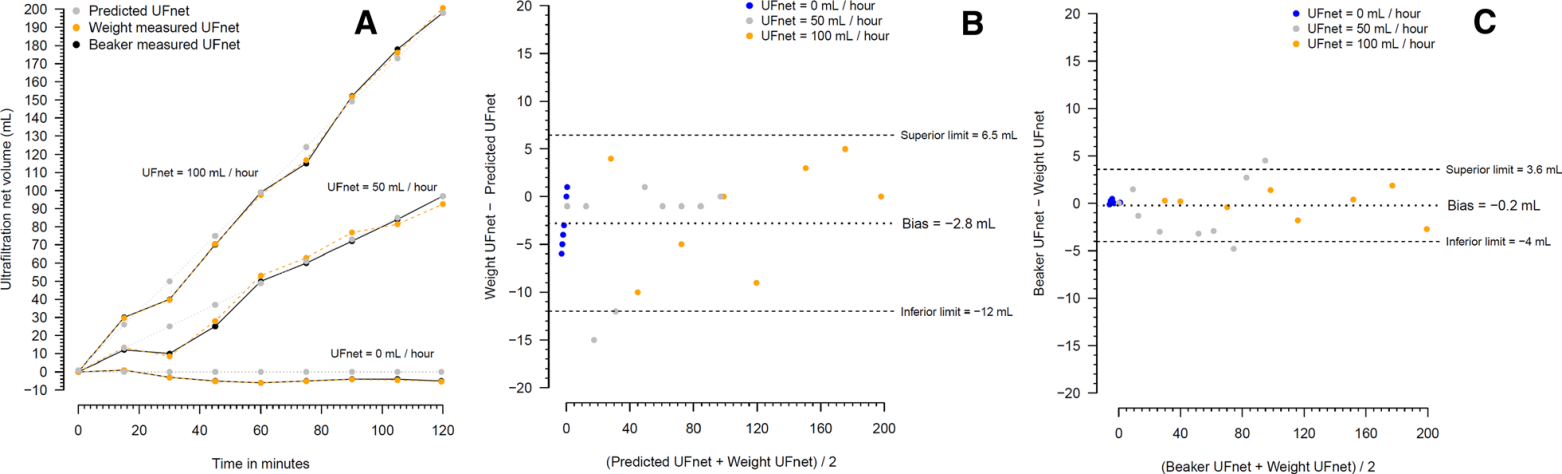
The analysis of 0, 50, 100 mL/h of ultrafiltration net using a total volume of affluent (dose) of 900, 900 and 500 mL/h, respectively, during 120 min. **A** Shows the predicted cumulative UF, the weight measured cumulative UF, and the beaker measured cumulative UF. **B** Shows the Bland–Altman diagram measuring the agreement of UFnet measured through the affluent and effluent weight difference and UFnet predicted. **C** Shows the Bland–Altman diagram measuring the agreement of UFnet measured through the beaker and through the affluent and effluent weight difference. UF denotes ultrafiltration rate. The affluent and effluent pumps worked intermittently, synchronized, and with variable bits output from microcontroller. Predicted cumulative UFnet was calculated dividing the set UFnet/60 min and adding this value each minute observed during the experiment. The configuration 2 of Additional file 1: Fig. S4 was used in this experiment

Figure 4 shows the linear cumulative UFnet over the 120 min of the experiment, using the preset UFnets of 0, 50, and 100 mL/h, as well as the correlation and agreement of the measured UFnet with the predicted UFnet. Additional file 1: Figure S28 shows the median number of bits output from the microcontroller to each pump during the 2 h of the experiment, according to the preset UFnet. In this figure, only bits when the motor pumps were on were accomplished. Additional file 1: Figure S29 shows the percentage of motor pumps on and off according to the UFnet preset.

### Experiment 5

**Fig. 5:**
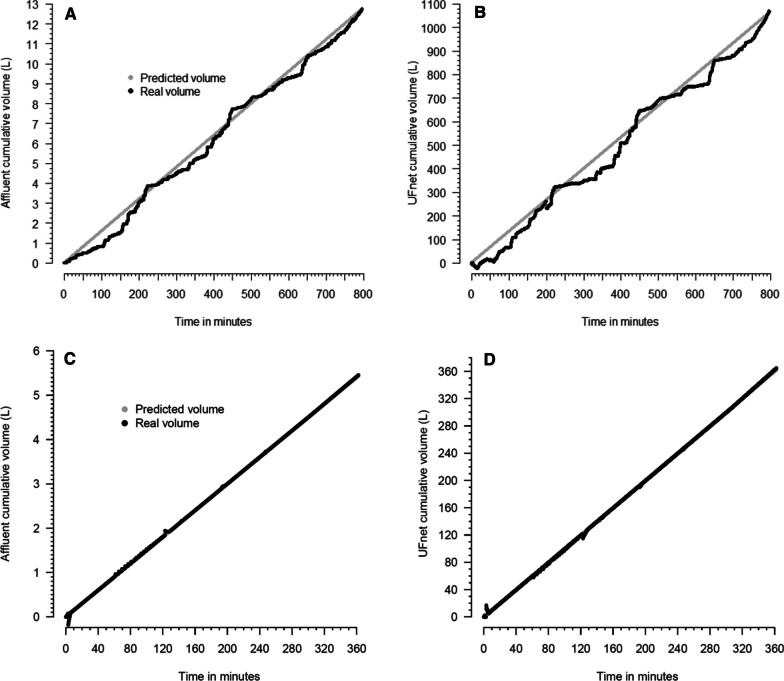
Two prolonged experiments data. **A**, **B** Show 20,396 data points collected during 797 min of cumulative affluent volume and cumulative ultrafiltration volume, respectively, as well as the predicted cumulative affluent and ultrafiltration volumes. In this experiment, an affluent volume of 900 mL/h and an ultrafiltration rate of 80 mL/h were set. **C**, **D** Show 9990 data points collected during 362 min of cumulative affluent volume and cumulative ultrafiltration volume, respectively, as well as the predicted cumulative affluent and ultrafiltration volumes. In this experiment, an affluent volume of 900 mL/h and an ultrafiltration rate of 60 mL/h were set. UF denotes ultrafiltration. The configuration 3 of Additional file 1: Fig. S4 was used in this experiment

Figure 5 shows the homogeneity of the cumulative affluent volume and cumulative UFnet over the time of the two prolonged experiments.

Additional file 1: Figure S30—Panel A shows the median number of bits of both pumps when the motors were on in the 797-min experiment. Additional file 1: Figure S30—Panel B shows the percentage of time with the motor on and off during this experiment. Additional file 1: Figure S31 shows the affluent fluid flow during the experiment. Additional file 1: Figure S32—Panel A shows the median number of bits of both pumps when the motors were on in the 362-min experiment. Additional file 1: Figure S32—Panel B shows the percentage of time with the motor on and off during this experiment. Additional file 1: Figure S33 shows the affluent fluid flow during the experiment.

## Discussion

In this study, we built a dialysate controller for experimental purposes with a primary fluid flow control based on the peristaltic pump principles, using the fluid bag weight variation as a backup to correct the fluid flow over time. Our results showed a reasonable precision of the affluent flow control, with a good correlation *R*^2^ = 0.99 for continuous flow and *R*^2^ = 0.95 for intermittent flow, and an acceptable agreement with a bias = 3.4 mL (upper limit 95% = 43.9 mL and lower limit 95% = − 37 mL) for continuous flow and bias = − 20.9 mL (upper limit 95% = 54 mL and lower limit 95% = − 95.7 mL) for intermittent flow. We also found a good precision of cumulative Ufnet value reached over time when compared to the predicted (calculated) UFnet, with a bias = − 2.8 mL (upper limit 95% = 6.5 mL and lower limit 95% = − 12 mL). These findings resulted from affluent flows as low as 8, 12, 20 mL/min, which are values compatible with 25–30 mL/kg of CRRT dose for medium animals such as swines, and this acceptable precision of our results was kept when the controller was used with a high filter dialysate chamber pressure for 797 min.

This study was a first step in building an experimental CRRT machine aiming to explore special situations in critically ill subjects with severe AKI. This device has a low assembling and maintenance cost, which is especially important when the resources are scarce, and when these resources must be directed for patients. The dialysate controller is the main step in this experimental process because the precision of affluent and UFnet flows must be highly calibrated for medium animals (20–40 kg swine for instance). About the potential clinical effect of UFnet errors, a UFnet higher than 1.75 mL/kg/h or lesser than 1.01 mL/kg/h is associated with a higher mortality in humans [26], raising the concern about UFnet errors and their consequences.

Current commercial dialysis machines can present UFnet errors as high as 25.1 ± 27.9 mL, although mean UFnet errors less than 30 mL/h are considered acceptable for some authors in the adult patient’s literature [27]. Additionally, in the validation of a high-precision CRRT machine for infants and children, the maximum UFnet error allowed was 1 g/h [28]. These values of error are potentially high for neonatal, pediatric, and medium/small subjects [28, 29]. The Food and Drug Administration Quality Assurance Guidelines for Hemodialysis Devices recommends an actual UFnet error lesser than 10% of the preset [30]. Our device reached a bias of − 2.8 mL in 2 h, which is acceptable.

One step toward the safety of a CRRT device use in small subjects is the reduction in the extracorporeal circuit, avoiding excessive hemodilution at the beginning of the support, avoiding high heat exchange, and extremely low flow regions inside the circuit, favoring the circuit and filter coagulation, especially during prolonged low blood flows [31, 32]. On the other hand, a low-volume extracorporeal circuit associated with low blood flow is not able to keep a high UFnet precision [33], probably for the low transmembrane pressure generated. For very low-weight neonates, a technical solution has been successfully reached with blood and dialysate pumped through syringes [29, 34]. Our device was built for blood flows of 75–120 mL/min (subjects with weight of 20–40 kg), which are compatible with blood flows used in adult patients with severe AKI care.

High pressures in the dialysis filter fluid compartment are associated with higher UFnet errors in many currently used CRRT machines [35]; however, an afferent and efferent fluid bag weight variation as a servo-controlled mechanism is associated with a very low error in UFnet in children [35, 36]. In this way, our device was able to compensate for a very high filter dialysate chamber pressure (150 mmHg).

Many bedside CRRT machines use stepper motors to drive the peristaltic pump [37]. These motors can increase the precision of the device [37]; however, the cost of a stepper motor is as high as 20 times the cost of a regular motor, which was used to build this device.

Some limitations must be discussed: (1) we have not measured the actual solute clearance efficiency in this experiment. Despite the acceptable precision of affluent and UFnet flows, the backfiltration effect can reduce the solute clearance efficiency due to the dynamics of affluent/effluent flow characteristics [21]. Nevertheless, in a previous similar experiment of our group, the fluid urea nitrogen/blood urea nitrogen (FUN/BUN) ratio, a CRRT measure of low molecular weight solute clearance efficiency [38], was high during the 12 h of dialysis experiment, denoting a high clearance capacity; (2) we have not tested the dialysate controller for periods higher 12 h, which can enhance the cumulative measured errors; however, the CRRT experiments are currently shorter than 12 h [6, 35].

## Conclusions

We developed and tested a dialysate controller with commendable precision in delivering the cumulative CRRT dose and maintaining a satisfactory level of precision in calculating the UFnet. This equipment is of a noteworthy very low cost, which allows its utilization for teaching and experimental purposes for the study of CRRT in low and middle-income countries.

### Supplementary Information

**Additional file 1.** Supplementary Table and Figures.

## Data Availability

The database is available for reasonable requests.
