# Supplementary material for: Validation of a low-cost continuous renal replacement therapy dialysate fluid controller for experimental purposes
Source: Intensive Care Med Exp. 2024 Feb 2;12:9. doi: 10.1186/s40635-024-00593-z (PMC10834914; doi:10.1186/s40635-024-00593-z)
Supplement: Supplementary file 1 — Additional file 1. Supplementary Table and Figures. [file 40635_2024_593_MOESM1_ESM.docx]

ADDITIONAL FILE 1

VALIDATION OF A LOW-COST CONTINUOUS RENAL REPLACEMENT THERAPY DIALYSATE FLUID CONTROLLER FOR EXPERIMENTAL PURPOSES.

Running title: Experimental low-cost dialysate controller validation

Yuri de Albuquerque Pessoa dos Santos,^1,2^ Viviane Flor Park,^3^ Luis Carlos Maia Cardozo Junior,^1,2^ Bruno Adler Maccagnan Pinheiro Besen,^1,2^ Pedro Vitale Mendes,^1,2^ and Marcelo Park.^1,2^

1. Medical Intensive Care Unit; Internal Medicine Division, Hospital das Clínicas HCFMUSP; Faculdade de Medicina, Universidade de São Paulo, São Paulo, SP, Brazil.
2. Laboratory of Medical Investigation (LIM-51); Emergency Discipline; Faculdade de Medicina, Universidade de São Paulo, São Paulo, SP, Brazil.
3. Institute of Science and Technology; Federal University of São Paulo; São José dos Campos; São Paulo; Brazil.

SUMMARY

[METHODS 5](#_Toc148315279)

[Table S1: Prices of each hardware component and total costs 5](#_Toc148315280)

[Figure S1: The final design of the controller device. 6](#_Toc148315281)

[Figure S2: Electrical/electronic circuit of the peristaltic pump motors power amplifiers. 7](#_Toc148315282)

[Figure S3: Electrical/electronic circuit of the strain gauge balances and load amplifiers used in order to weight the fluid bags, through the microcontroller. 8](#_Toc148315283)

[Figure S4: The different configurations of the dialysate controller used during the experiments. 9](#_Toc148315284)

[The final sketch 12](#_Toc148315285)

[RESULTS 19](#_Toc148315286)

[Figure S6: Correlation between the microcontroller output in bits and the resultant power amplifier potential difference generated to the pump motors. 19](#_Toc148315287)

[Figure S7: Correlation between the power amplifier potential difference output in Volts and the resultant fluid flow, measured through the volume quantification using a beaker. 20](#_Toc148315288)

[Figure S8: Pump 2 working continuously with 120 bits of output from the microcontroller and transferring 500 mL of normal saline from balance 1 to balance 2. 21](#_Toc148315289)

[Figure S9: Pump 2 working continuously with 90 bits of output from the microcontroller and transferring 500 mL of normal saline from balance 1 to balance 2. 22](#_Toc148315290)

[Figure S10: Pump 2 working continuously with 80 bits of output from the microcontroller and transferring 500 mL of normal saline from balance 1 to balance 2. 23](#_Toc148315291)

[Figure S11: Pump 2 working continuously with 75 bits of output from the microcontroller and transferring 500 mL of normal saline from balance 1 to balance 2. 24](#_Toc148315292)

[Figure S12: Pump 2 working continuously with 60 bits of output from the microcontroller and transferring 500 mL of normal saline from balance 1 to balance 2. 25](#_Toc148315293)

[Figure S13: Pump 2 working continuously with 40 bits of output from the microcontroller and transferring 500 mL of normal saline from balance 1 to balance 2. 26](#_Toc148315294)

[Figure S14: Pump 2 working continuously with 35 bits of output from the microcontroller and transferring 500 mL of normal saline from balance 1 to balance 2. 27](#_Toc148315295)

[Figure S17: Fluid flow calculated trough the beaker measured volume beyond the time. Legend shows the bits outflow from the microcontroller. 30](#_Toc148315296)

[Figure S18: Correlation of beaker measured flow and weight measured flow retrieved from both balances (affluent and effluent fluids). 31](#_Toc148315297)

[Figure S19: Bland – Altman diagram agreement between beaker measured flow (volume) and weight measured flow using both balances (affluent and effluent). 32](#_Toc148315298)

[Figure S20: Pump 2 working intermittently (approximately 4 seconds on and 4 seconds off) with 10 bits of output from the microcontroller and transferring 500 mL of normal saline from balance 1 to balance 2. 33](#_Toc148315299)

[Figure S21: Pump 2 working intermittently (approximately 4 seconds on and 4 seconds off) with 5 bits of output from the microcontroller and transferring 500 mL of normal saline from balance 1 to balance 2. 34](#_Toc148315300)

[Figure S22: Fluid flow calculated trough the beaker measured volume beyond the time, using intermittent flow. 35](#_Toc148315301)

[Figure S23: Fluid flow measured through the fluid weight beyond the time, using variable intermittent value of bits and flow. 36](#_Toc148315302)

[Figure S24: Histogram showing the bits output from the microcontroller when the experiment was set to intermittent flow of 8 mL/minute. 37](#_Toc148315303)

[Figure S25: Histogram showing the bits output from the microcontroller when the experiment was set to intermittent flow of 12 mL/minute. 38](#_Toc148315304)

[Figure S26: Histogram showing the bits output from the microcontroller when the experiment was set to intermittent flow of 20 mL/minute. 39](#_Toc148315305)

[Figure S27: Pump 1 working intermittently (approximately 4 seconds on and 4 seconds off) with variable bits of output from the microcontroller and transferring a preset flow of 20 (gray and blue curves), 12 (green and yellow curves) and 8 (red and orange curves) mL/minute of normal saline from balance 1 to balance 2. 40](#_Toc148315306)

[Figure S28: Boxplot showing the median, P25^th^, P75^th^, minimum and maximum number of bits output from microcontroller during the two hours of experiment. 41](#_Toc148315307)

[Figure S29: Bar plot the percentage of the time during the two hours of experiment with pumps on and off. 42](#_Toc148315308)

[Figure S30: Data collected during the first prolonged bench experiment. 43](#_Toc148315309)

[Figure S31: The affluent flow over the 797 minutes observed during the first prolonged bench experiment. 44](#_Toc148315310)

[Figure S32: Data collected during the second prolonged bench experiment. 45](#_Toc148315311)

[Figure S33: The affluent flow over the 362 minutes observed during the first prolonged bench experiment. 46](#_Toc148315312)

# METHODS

## Table S1: Prices of each hardware component and total costs

| Arduino UNO R3 - 8.78 (US Dollars – $) USD. |
| --- |
| Two strain gauges - 5.56 USD each one. |
| Two HX711 amplifiers - 1.33 USD each one. |
| Three peristaltic pumps 12 Volts - 7.22 USD each one. |
| One protoboard + Jumpers - 5.17 USD. |
| Three TIP 120 transistors - 0.43 USD each one. |
| Three 1N 4002 diodes - 0.04 USD each one. |
| Three 200 ohms resistors - 0,01 USD each one. |
| One power electrical source of 12 Volts and 500 mA - 3.09 USD. |
| **Total cost: 53.87 USD** |


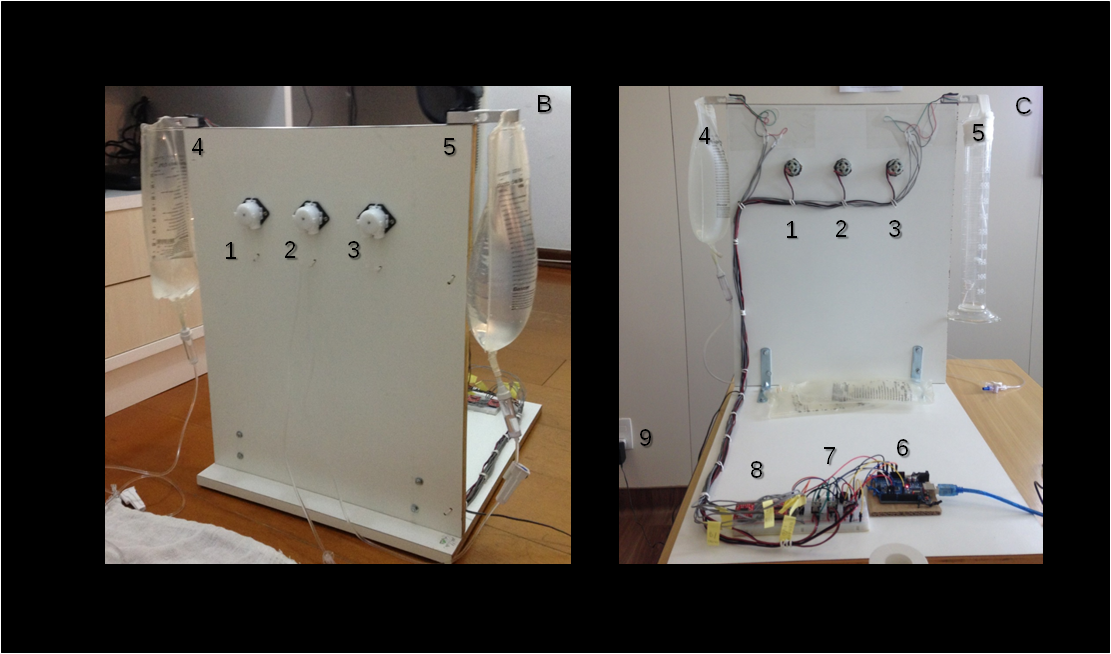
 Figure S1: The schematic view and final design of the controller device.

Panel A shows the schematic view

The arrows directions show the information or electrical current flow.

The buzzer was used to alarm twice, when the residual affluent volume was lower than 100 and 50 mL.

Panel B shows the front view and Panel C shows the back view.

The components of the controller device are identified according to the number below:

1. Peristaltic pump 1 – effluent fluid driver.
2. Peristaltic pump 2 – affluent fluid driver.
3. Peristaltic pump 3 – heparin driver.
4. Strain gauge 1 – effluent fluid bag balance.
5. Strain gauge 2 – affluent fluid bag balance.*
6. Arduino Uno.
7. Protoboard sector with three TIP 120 based power amplifier.
8. Protoboard sector with two HX711 strain gauge load amplifier.
9. Power 13 Volts 500 mA electrical source.

* Panel B shows the graduated cylinder (Beaker) used as a bag to measure the volume and weight of fluids.

## Figure S2: Electrical/electronic circuit of the peristaltic pump motors power amplifiers.

Panel A shows the circuit and microcontroller’s analogical port connection. Panel B shows the Darlington’s disposition transistor TIP 120 used in the circuit in order to optimize the pumps power source. Each of the three peristaltic pump motors has an individual electrical power source amplifier.

Figure S3: Electrical/electronic circuit of the strain gauge balances and load amplifiers used in order to weight the fluid bags, through the microcontroller.

Each of the two balances has an individual load amplifier.

V denotes the potential difference in the Wheatstone bridge, the degree of this potential difference is linearly correlated with the weight of the structure that deformed the strain gauge.

## Figure S4: The different configurations of the dialysate controller used during the experiments.

Configuration 1 was used to validate the transference of fluid from balance 1 (affluent) to balance 2 (effluent). Attention to the disposition of beaker hanging on the balance, in order to measure the volume and weight of effluent fluid.

Configuration 2 was used to validate the ultrafiltration rate using two pumps and two balances. The beaker was used as intermediary in which the affluent fluid enter into the cylinder and the effluent was aspirated from the cylinder onto the bag of balance 2 through the pump 2. The behavior of the beaker was as the patient in terms of fluid balance.

Configuration 3 was used as the configuration 2; however, a pressurized bag (with 100 mmHg) was used instead of beaker, simulating the external compartment of a high flow metabolic filter (dialysis filter) with a high inlet pressureand low transmembrane pressure. This configuration was used with the 6 – 12 hours experiments.

Please find below the legend explaining each component of the three configurations:

Figure S5: Sketch structure of the microcontroller, trying to keep a stable affluent flow and UF rate. UF denotes ultrafiltration rate.

## The final sketch

// motores portas 3/6/5

#include <HX711.h>

#define pinDT1 A0

#define pinSCK A5

#define pinDT2 A2

HX711 bala1; HX711 bala2;

float fluxo; int uf = 0; int aflu = 1000; int seg = 0;

int a = 0; int controle = 0; int hep = 0; int aftot = 0;

float medidab1 = 0; float medidab2 = 0; float medseg;

float medidab1bk; float medidab2bk;

float medida_inib1 = 0; float medida_inib2 = 0;

float bits; float bits2; float bits4;

long tempoinicio = 0; long tempodec = 0; long tempohep;

float vpreditoaf; float vrealaf; float erroaf = 0; float erroafp;

float vpreditoef; float vrealef; float erroef = 0; float erroefp; float ufpred = 0; float ufreal = 0;

void setup() {

pinMode(3, OUTPUT); pinMode(6, OUTPUT); pinMode(5, OUTPUT); pinMode(10,OUTPUT);

Serial.begin(9600);

Serial.println("Qual o volume de afluente em mL por hora?: 9 - diminui e 0 aumenta/ao terminar pressione 8 (Sempre seguido de enter)");

quant_aflu:

Serial.print("Valor do afluente = ");

Serial.println (aflu);

while(1){if (Serial.available()) {char caractere = Serial.read(); controle = caractere - 48; if (controle == 8){goto segue_prog;} break;}}

if (controle == 0){aflu = aflu + 50;}

if (controle == 9){aflu = aflu - 50;}

goto quant_aflu;

segue_prog:

Serial.println(""); Serial.println("Qual o volume de ultrafiltrado em mL por hora?: 9 - diminui e 0 aumenta/ao terminar pressione 8 (Sempre seguido de enter)");

quant_uf:

Serial.print("Valor de UF = ");

Serial.println (uf);

while(1){if (Serial.available()) {char caractere = Serial.read(); controle = caractere - 48; if (controle == 8){goto segue_prog1;} break;}}

if (controle == 0){uf = uf + 5;}

if (controle == 9){uf = uf - 5;}

goto quant_uf;

segue_prog1:

// Calculo de bits

bits = round(((aflu/60) - 0.67)/0.7);

Serial.println(""); Serial.println ("Mantenha as balanças livres, digite zero e enter para inicialização");

while (1){if (Serial.read () == 48) {break;}}

Serial.println(""); Serial.println("Inicializando .................................................................................................................................");

bala1.begin(pinDT1, pinSCK); bala1.set_scale(226181); bala2.begin(pinDT2, pinSCK); bala2.set_scale(-203386);

bala1.tare(); bala2.tare();

bala1.power_down(); bala2.power_down(); delay(2000);

bala1.power_up(); bala1.tare(); bala1.power_down(); delay(2000);

bala2.power_up(); bala2.tare(); bala2.power_down();

Serial.println(""); Serial.println("Sistema inicializado - pode pendurar as soluções!");

Serial.println(""); Serial.println("Digite 0 para modificar a taxa de UF");

Serial.println(""); Serial.println("Digite 1 para iniciar ou reiniciar a terapia");

Serial.println(""); Serial.println("Digite 2 para parar a terapia");

Serial.println(""); Serial.println("Digite 3 para desligar o alarme");

Serial.println(""); Serial.println("Digite 4 para ligar a heparina");

Serial.println(""); Serial.println("Digite 5 para flush afluente quando em pausa");

Serial.println(""); Serial.println("Digite 6 para flush efluente quando em pausa");

Serial.println(""); Serial.println("Digite 7 para flush de heparina quando em pausa");

Serial.println(""); Serial.println("Digite 8 para parar flush");

Serial.println(""); Serial.println("Digite 9 para parar heparina");

Serial.println(""); Serial.println("##################### Sempre seguido de enter!!!! ####################");Serial.println();}

void loop() {

if (Serial.available()) {char caractere = Serial.read(); controle = caractere - 48;}

if ((controle == 1) && (a == 0)) {

pesoinicialb1:

check_bala1();

if((medidab1 - medidab1bk < - 0.01) || (medidab1 - medidab1bk > 0.01)) {goto pesoinicialb1;}

medida_inib1 = medidab1; if (bits < 40){bits2 = 50;} else {bits2 = bits;}

pesoinicialb2:

check_bala2();

if((medidab2 - medidab2bk < - 0.030) || (medidab2 - medidab2bk > 0.01)) {goto pesoinicialb2;}

medida_inib2 = medidab2; a = 1; if (bits < 60){bits4 = 65;} else {bits4 = bits;} analogWrite(5, bits4); analogWrite(6, bits2); tempoinicio = millis();

tempohep = tempoinicio;}

tempodec = millis() - tempoinicio;

bala1med:

check_bala1();

if (a != 0){if ((medidab1 - medidab1bk < - 0.01) || (medidab1 - medidab1bk > 0.01)){delay(50); goto bala1med;}}

bala2med:

check_bala2();

if (a != 0){if ((medidab2 - medidab2bk < - 0.025) || (medidab2 - medidab2bk > 0.02)){if (seg >= 3){medidab2 = medseg;} seg = seg + 1; delay(50); goto bala2med;}}

medseg = medidab2;

vpreditoaf = (aflu/60) * tempodec/60000;

vpreditoef = ((aflu + uf + 80)/60) * tempodec/60000;

vrealaf = ((medida_inib1 - medidab1) * 1000);

vrealef = (medidab2 - medida_inib2) * 1000;

ufpred = vpreditoef - vpreditoaf;

ufreal = vrealef - vrealaf;

if (a != 0 ){ajustes_fluxoaf (); ajustes_fluxoef ();} else {vpreditoaf = 0; vrealaf = 0; vpreditoef = 0; vrealef = 0; ufpred = 0; ufreal = 0;}

serial_output ();

if (hep == 1){if (a != 0 && tempodec > tempohep + (10*60000)){tempohep = tempodec; Serial.println("Dose de heparina");digitalWrite(3,60);delay(1000);digitalWrite(3,0);}}

if (controle == 0 && a != 0){

Serial.println(""); Serial.println("Qual o volume de ultrafiltrado em mL por hora?: 9 - diminui e 0 aumenta/ao terminar pressione 8 (Sempre seguido de enter)");

quant_uf1:

Serial.print("Valor de UF = ");

Serial.println (uf);

while(1){if (Serial.available()) {char caractere = Serial.read(); controle = caractere - 48; if (controle == 8){controle = 1; goto segue_prog2;} break;}}

if (controle == 0){uf = uf + 5;}

if (controle == 9){uf = uf - 5;}

goto quant_uf1;}

segue_prog2:

troca:

if (controle == 2 && a != 0){analogWrite(3, 0); analogWrite(5, 0); analogWrite(6, 0); digitalWrite(10,0);

pausa:

while(1){if (Serial.available()) {char caractere = Serial.read(); controle = caractere - 48; if (controle == 1){

analogWrite(5, 0); analogWrite(3, 0); analogWrite(6, 0); a = 0; aftot = aftot + vrealaf; goto pospausa;} break;}}

if (controle == 5){analogWrite(6, 255);}

if (controle == 6){analogWrite(5, 255);}

if (controle == 7){analogWrite(3, 255);}

if (controle == 8){analogWrite(3, 0); analogWrite(5, 0); analogWrite(6, 0);}

goto pausa;

pospausa:;}

if (controle == 4) {Serial.println ("Infusao de heparina acionada"); controle = 1; hep = 1;}

if (controle == 9) {digitalWrite(3,0); Serial.println ("Infusao de heparina desligada"); hep = 0;}

if (controle == 3) {digitalWrite(10,0); goto fim;}

if (medidab1 * 1000 < 60 && a != 0) {digitalWrite(10,1); delay(200);digitalWrite(10,0);}

if (medidab1 * 1000 < 55 && a != 0) {digitalWrite(10,0); analogWrite(5, 0); analogWrite(3, 0); analogWrite(6, 0);

Serial.println("");Serial.println("Troque o afluente"); controle = 2; goto troca;}

fim:;}

void check_bala1 (){

bala1.power_up();

delay(200);

medidab1 = bala1.get_units(10);

medidab1bk = bala1.get_units(10);

bala1.power_down();}

void check_bala2 (){

bala2.power_up();

medidab2 = bala2.get_units(20);

bala2.power_down();

bala2.power_up();

medidab2bk = bala2.get_units(20);

bala2.power_down();}

void serial_output (){

Serial.print ("> t (seg) -> ");

Serial.print (tempodec/1000);

Serial.print (" > Afluente total = ");

Serial.print (aftot + vpreditoaf,0);

//Serial.print (" > predito af = ");

//Serial.print (vpreditoaf,0);

Serial.print (" > real af = ");

Serial.print (vrealaf,0);

//Serial.print (" > predito ef -> ");

//Serial.print (vpreditoef,0);

Serial.print (" ");

Serial.print ("> real ef -> ");

Serial.print (vrealef- ((110/60)*(tempodec/60000)),0);

Serial.print (" ");

Serial.print ("> UF predita -> ");

Serial.print (ufpred - ((110/60)*(tempodec/60000)),0);

Serial.print (" ");

Serial.print ("> UF real -> ");

Serial.print (ufreal - ((110/60)*(tempodec/60000)),0);

Serial.print (" > bits (2/4) = ");

Serial.print (bits2,0);

Serial.print ("/");

Serial.println (bits4,0);}

void ajustes_fluxoaf (){

erroafp = erroaf;

erroaf = vrealaf - vpreditoaf;

if (tempodec < 300000){

if (erroaf <= 2 && erroaf >= -2 && erroafp > erroaf && bits2 != 0){bits2 = bits2 + 10; goto fim_ajustes;}

if (erroaf <= 2 && erroaf >= -2 && erroafp < erroaf && bits2 != 0){bits2 = bits2 - 10; goto fim_ajustes;};}

if (erroaf <= 2 && bits2 == 0) {bits2 = 50; goto fim_ajustes;}

if (erroaf > 12) {bits2 = 0; goto fim_ajustes;}

if (erroaf > 8) {bits2 = bits2 - 20; goto fim_ajustes;}

if (erroaf > 6) {bits2 = bits2 - 10; goto fim_ajustes;}

if (erroaf > 2) {bits2 = bits2 - 5; goto fim_ajustes;}

if (erroaf < - 12) {bits2 = bits2 + 150; goto fim_ajustes;}

if (erroaf < - 8) {bits2 = bits2 + 20; goto fim_ajustes;}

if (erroaf < - 6) {bits2 = bits2 + 10; goto fim_ajustes;}

if (erroaf < - 2) {bits2 = bits2 + 5; goto fim_ajustes;}

fim_ajustes:;

if (bits2 <= 40){bits2 = 0;}

if (bits2 >= 255){bits2 = 255;}

analogWrite(6, bits2);}

void ajustes_fluxoef (){

erroefp = erroef;

erroef = ufreal - ufpred;

if (erroef < - 10) {bits4 = bits4 + 60; goto fim_ajustese;}

if (erroef <= 2 && bits4 == 0){bits4 = 70; goto fim_ajustese;}

if (erroef <= 2 && erroef >= -2 && erroefp < erroef && bits4 > 100){bits4 = bits4 - 25; goto fim_ajustese;}

if (erroef > 10) {bits4 = 0; goto fim_ajustese;}

if (erroef > 8) {bits4 = bits4 - 20; goto fim_ajustese;}

if (erroef < - 8) {bits4 = bits4 + 25; goto fim_ajustese;}

if (erroef < - 6) {bits4 = bits4 + 15; goto fim_ajustese;}

if (erroef > 6) {bits4 = bits4 - 10; goto fim_ajustese;}

if (erroef < - 4) {bits4 = bits4 + 12; goto fim_ajustese;}

if (erroef > 4) {bits4 = bits4 - 8; goto fim_ajustese;}

if (erroef < - 2) {bits4 = bits4 + 4; goto fim_ajustese;}

if (erroef > 2) {bits4 = bits4 - 4; goto fim_ajustese;}

fim_ajustese:;

if (bits4 <= 65){bits4 = 0;}

if (bits4 >= 255){bits4 = 255;}

analogWrite(5, bits4);}

# RESULTS


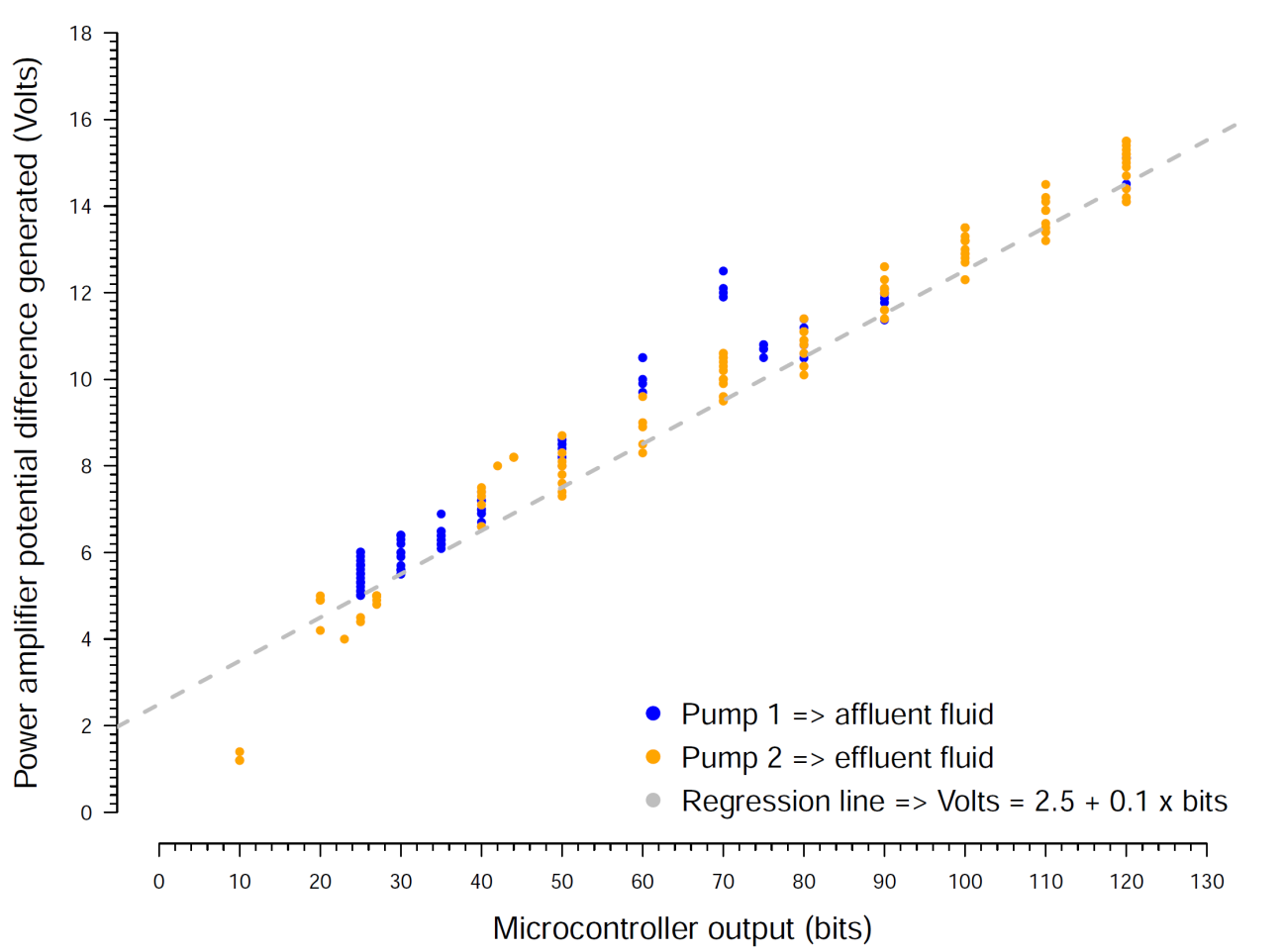


Figure S6: Correlation between the microcontroller output in bits and the resultant power amplifier potential difference generated to the pump motors.

The pump 1 (affluent) and pump 2 (effluent) were tested, with 120 and 100 timepoints respectively. The determinant coefficient measured was R^2^ = 0.78.


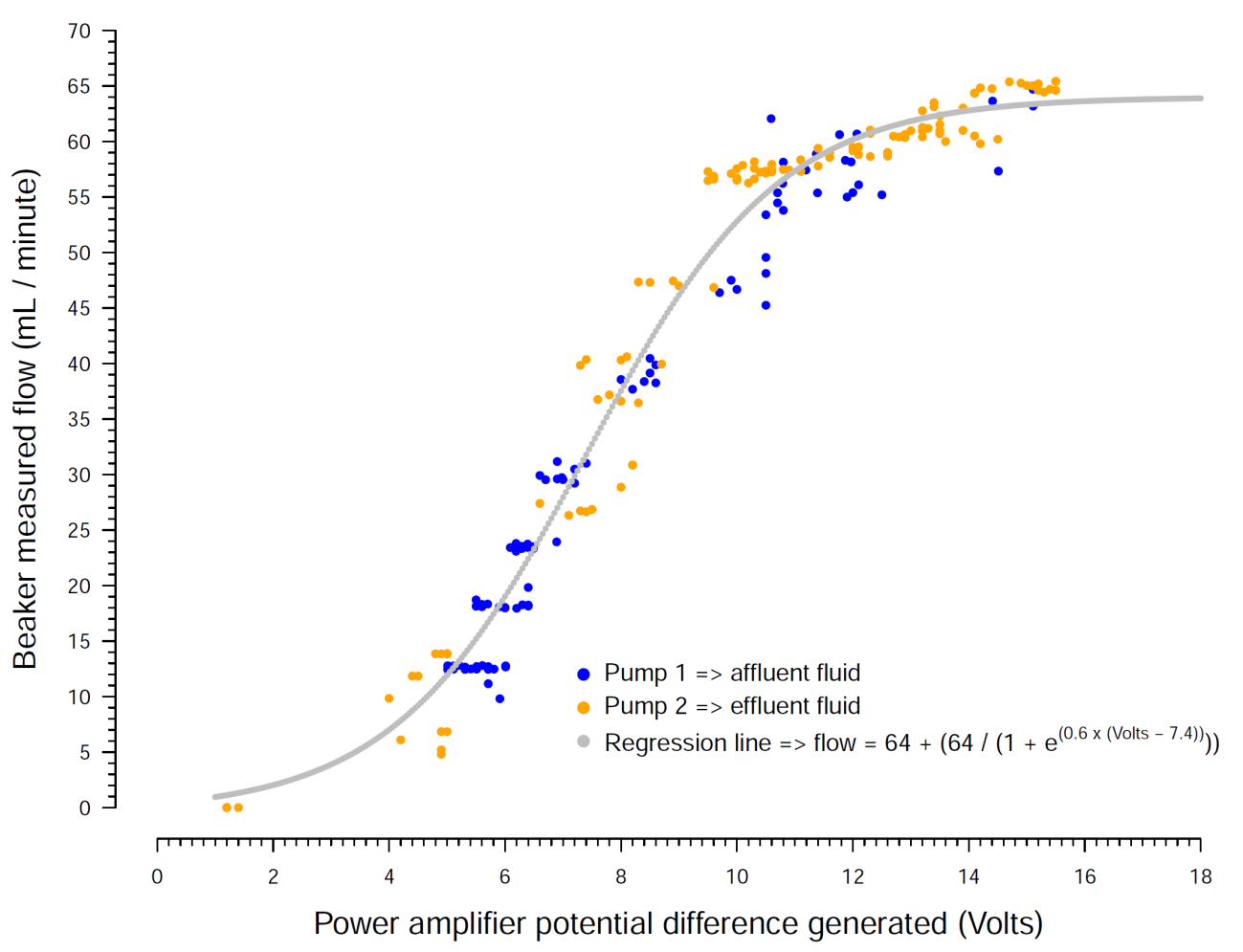


## Figure S7: Correlation between the power amplifier potential difference output in Volts and the resultant fluid flow, measured through the volume quantification using a beaker.

The pump 1 (affluent) and pump 2 (effluent) were tested, with 120 and 100 timepoints respectively.


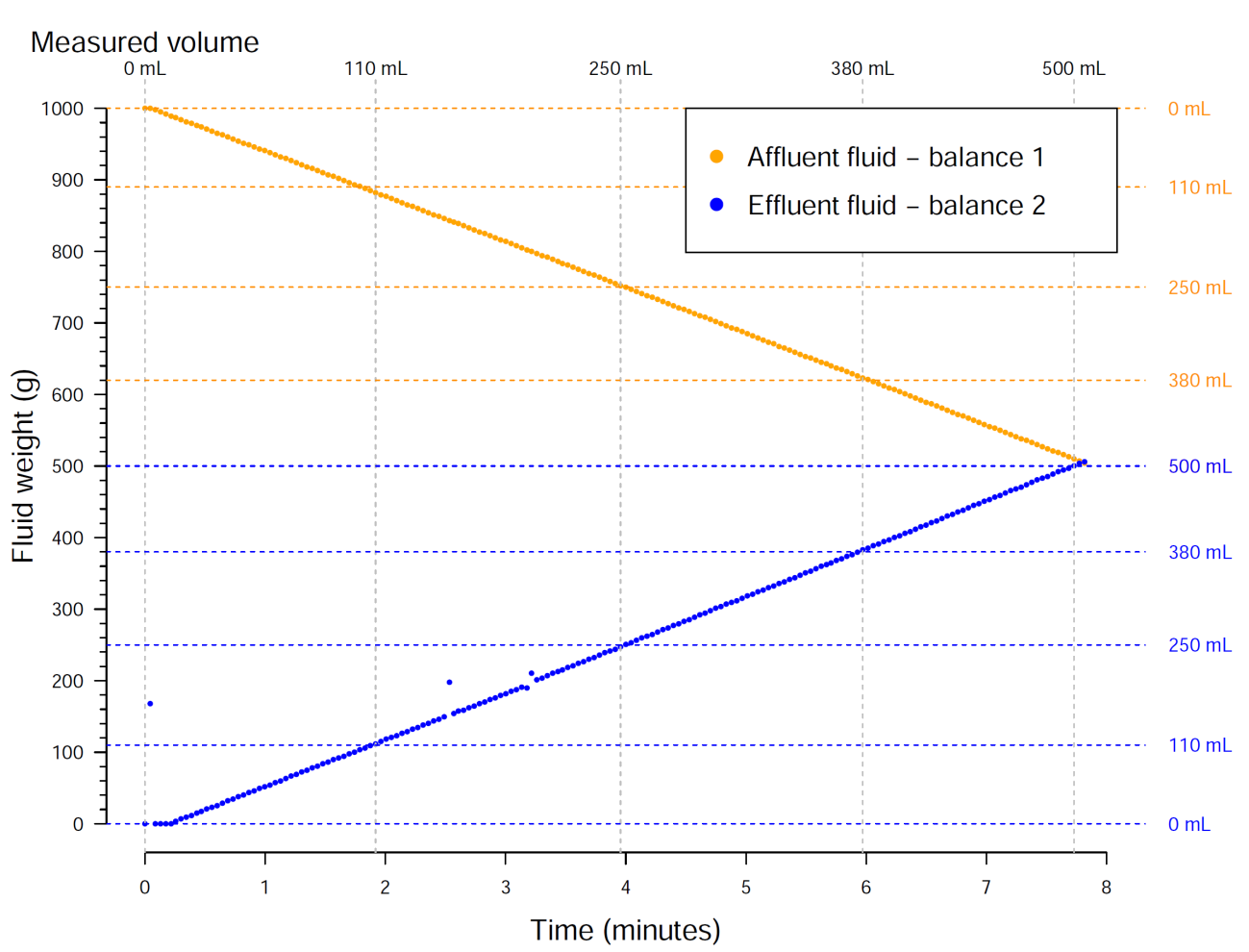


## Figure S8: Pump2working continuously with 120 bits of output from the microcontroller and transferring 500 mL of normal saline from balance 1 to balance 2.

We collected 180 timepoints values of fluid weight from each balance.

We collected the real normal saline volume transferred from balance 1 to balance 2 each approximately 2 minutes (Presented as measured volume).

The time spent to complete the fluid transfer was 7.82 minutes.


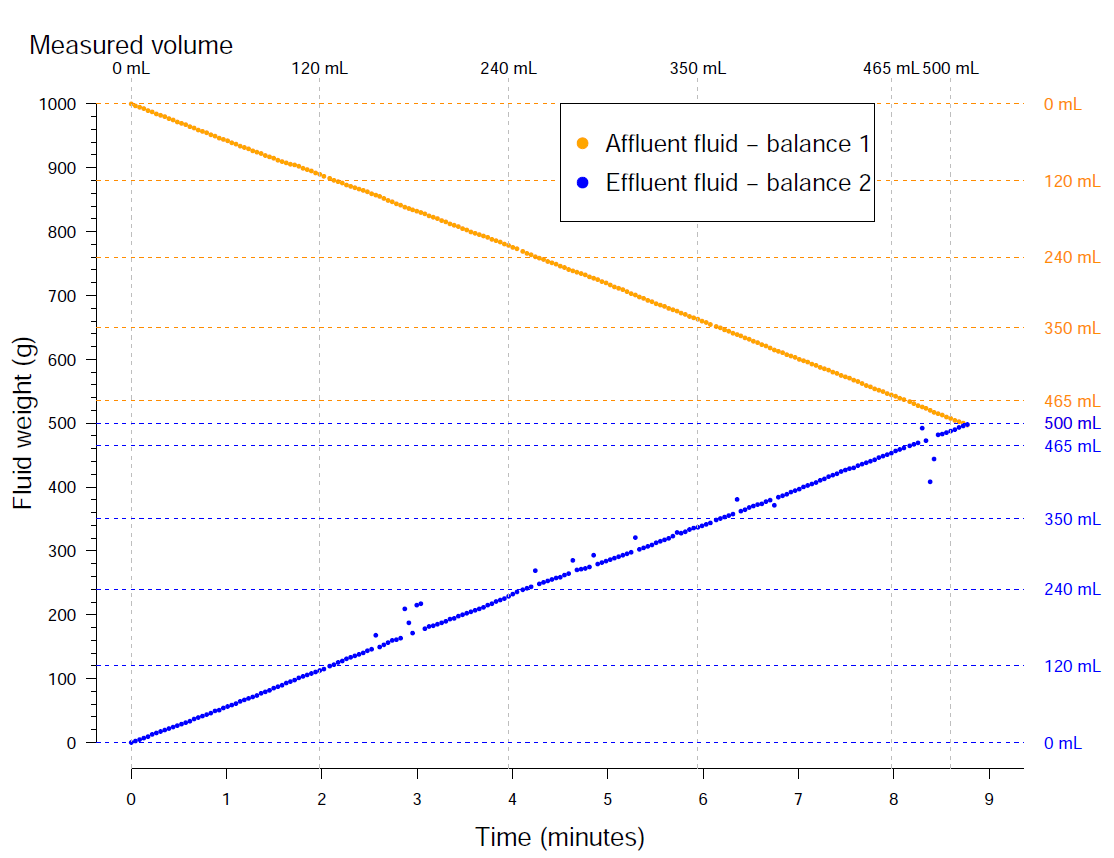


## Figure S9: Pump 2 working continuously with 90 bits of output from the microcontroller and transferring 500 mL of normal saline from balance 1 to balance 2.

We collected 200 time points values of fluid weight from each balance.

We collected the real normal saline volume transferred from balance 1 to balance 2 each approximately 2 minutes (Presented as measured volume).

The time spent to complete the fluid transfer was 8.77 minutes.


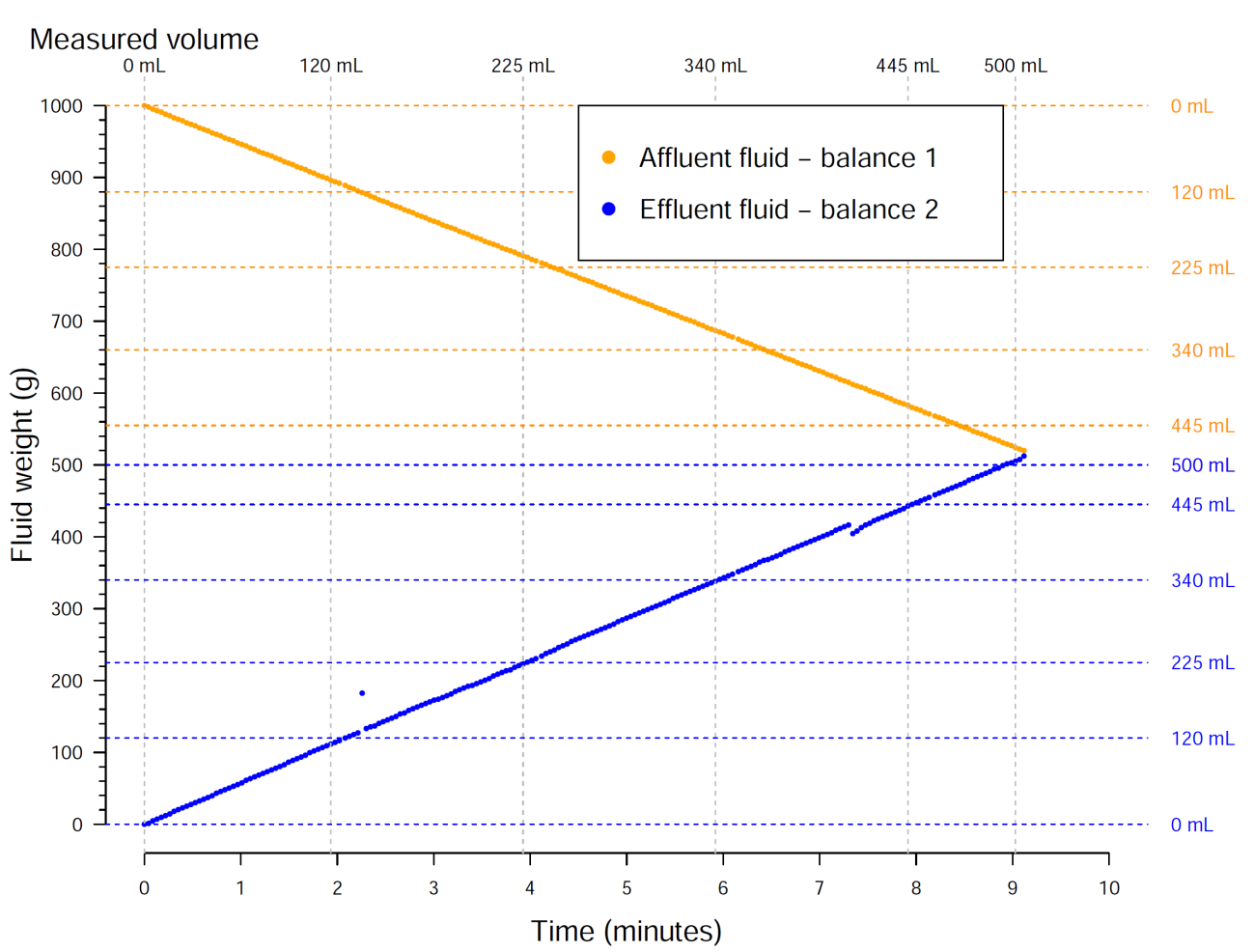


## Figure S10: Pump2 working continuously with 80 bits of output from the microcontroller and transferring 500 mL of normal saline from balance 1 to balance 2.

We collected 207 timepoints values of fluid weight from each balance.

We collected the real normal saline volume transferred from balance 1 to balance 2 each approximately 2 minutes (Presented as measured volume).

The time spent to complete the fluid transfer was 9.12 minutes.


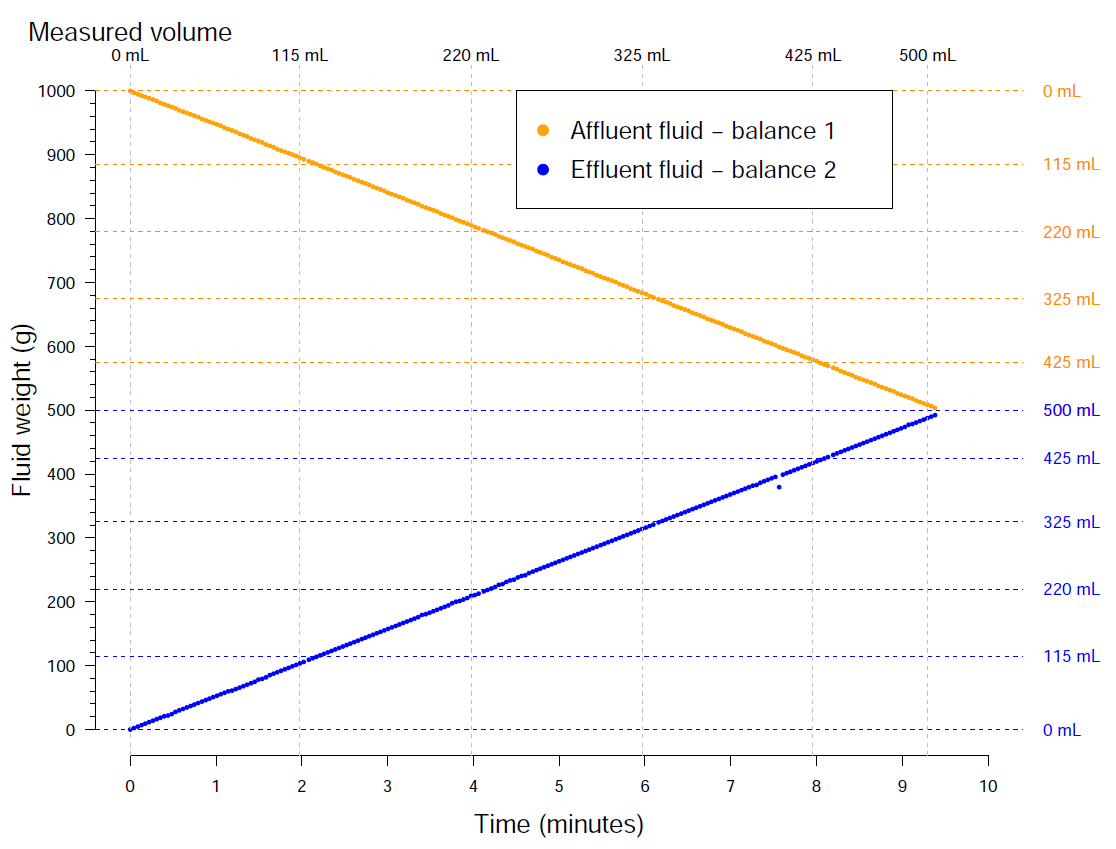


## Figure S11: Pump2 working continuously with 75 bits of output from the microcontroller and transferring 500 mL of normal saline from balance 1 to balance 2.

We collected 213 timepoints values of fluid weight from each balance.

We collected the real normal saline volume transferred from balance 1 to balance 2 each approximately 2 minutes (Presented as measured volume).

The time spent to complete the fluid transfer was 9.38 minutes.


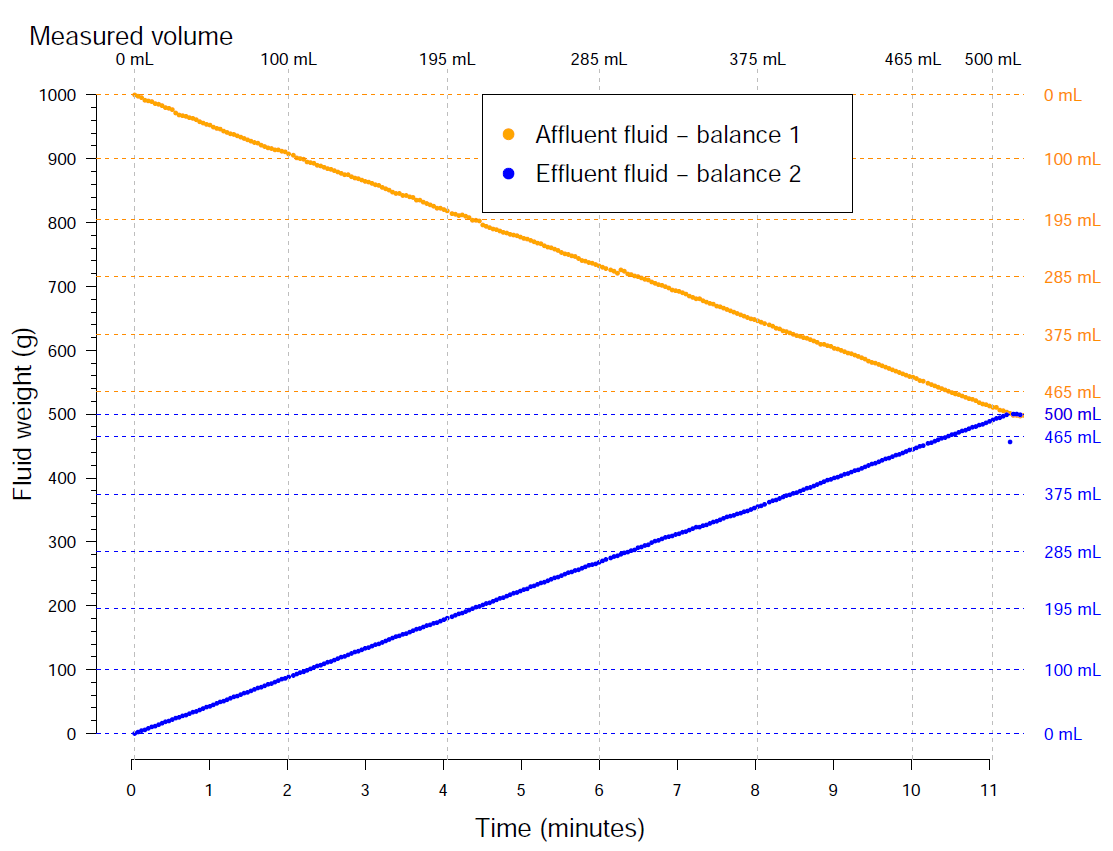


## Figure S12: Pump2 working continuously with 60 bits of output from the microcontroller and transferring 500 mL of normal saline from balance 1 to balance 2.

We collected 268 timepoints values of fluid weight from each balance.

We collected the real normal saline volume transferred from balance 1 to balance 2 each approximately 2 minutes (Presented as measured volume).

The time spent to complete the fluid transfer was 11.2 minutes.


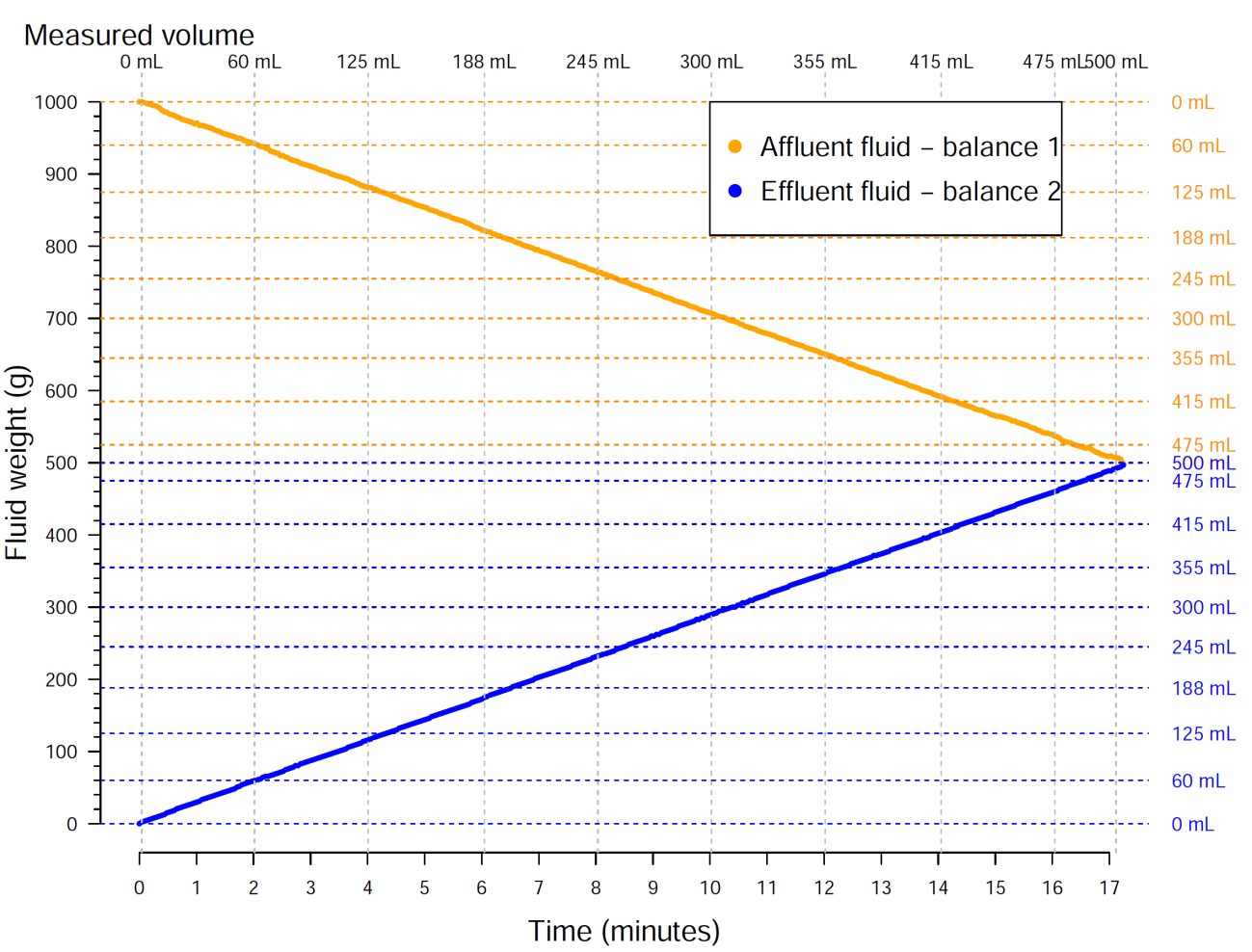


## Figure S13: Pump2 working continuously with 40 bits of output from the microcontroller and transferring 500 mL of normal saline from balance 1 to balance 2.

We collected 391 timepoints values of fluid weight from each balance.

We collected the real normal saline volume transferred from balance 1 to balance 2 each approximately 2 minutes (Presented as measured volume).

The time spent to complete the fluid transfer was 17.2 minutes.


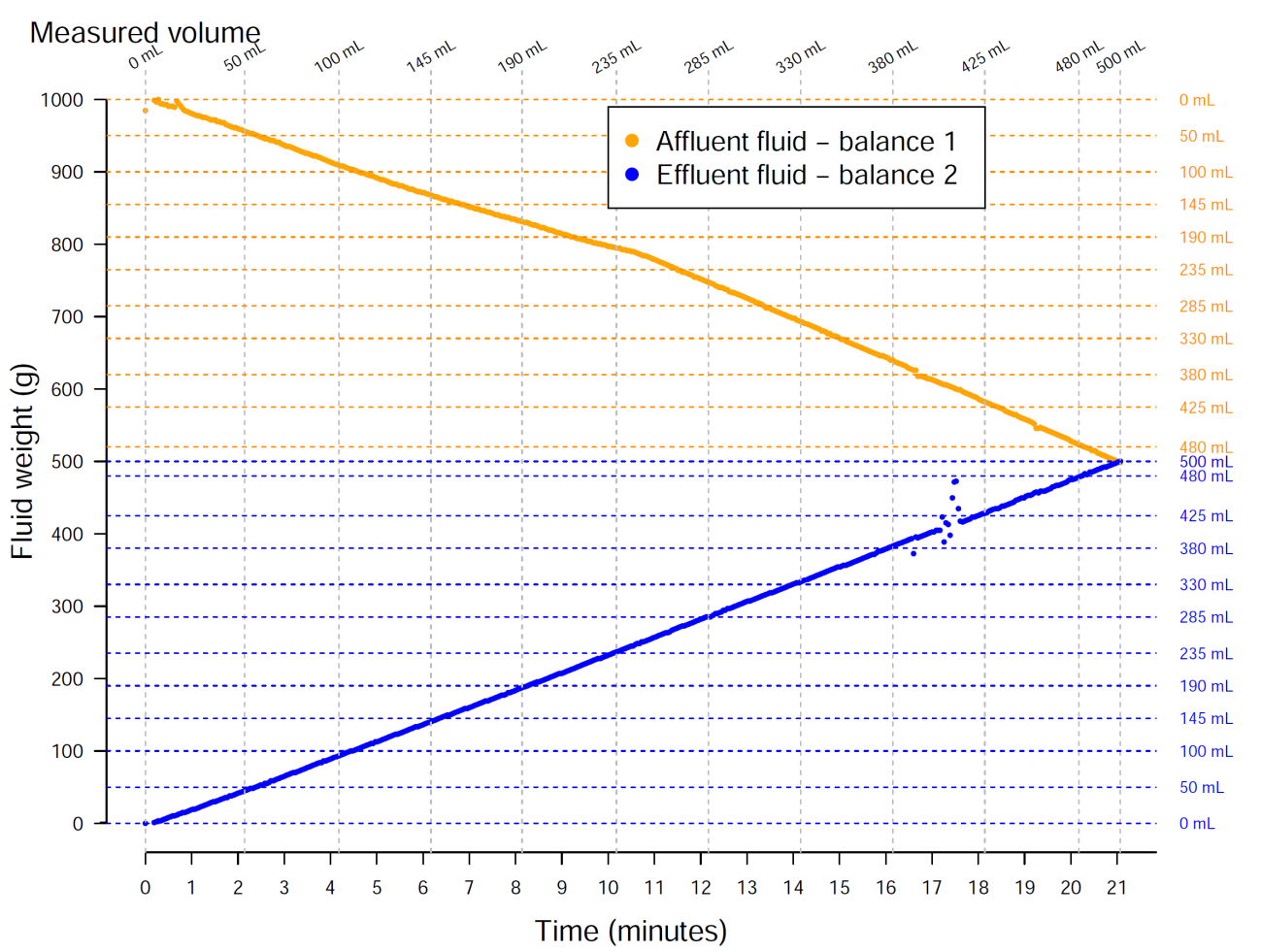


## Figure S14: Pump2 working continuously with 35 bits of output from the microcontroller and transferring 500 mL of normal saline from balance 1 to balance 2.

We collected 478 timepoints values of fluid weight from each balance.

We collected the real normal saline volume transferred from balance 1 to balance 2 each approximately 2 minutes (Presented as measured volume).

The time spent to complete the fluid transfer was 21.07 minutes.


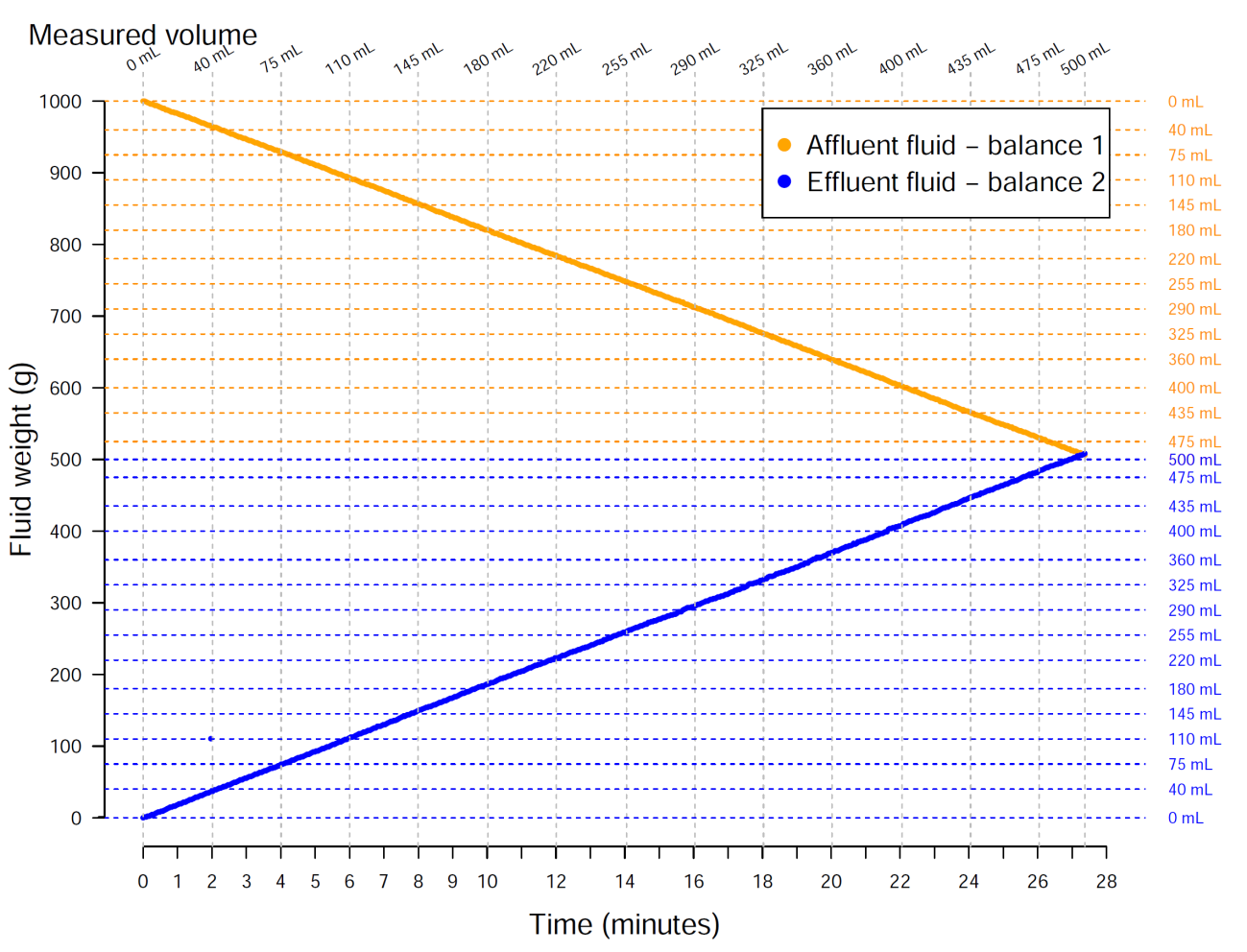


Figure S15: Pump2 working continuously with 30 bits of output from the microcontroller and transferring 500 mL of normal saline from balance 1 to balance 2.

We collected 620 timepoints values of fluid weight from each balance.

We collected the real normal saline volume transferred from balance 1 to balance 2 each approximately 2 minutes (Presented as measured volume).

The time spent to complete the fluid transfer was 27.37 minutes.


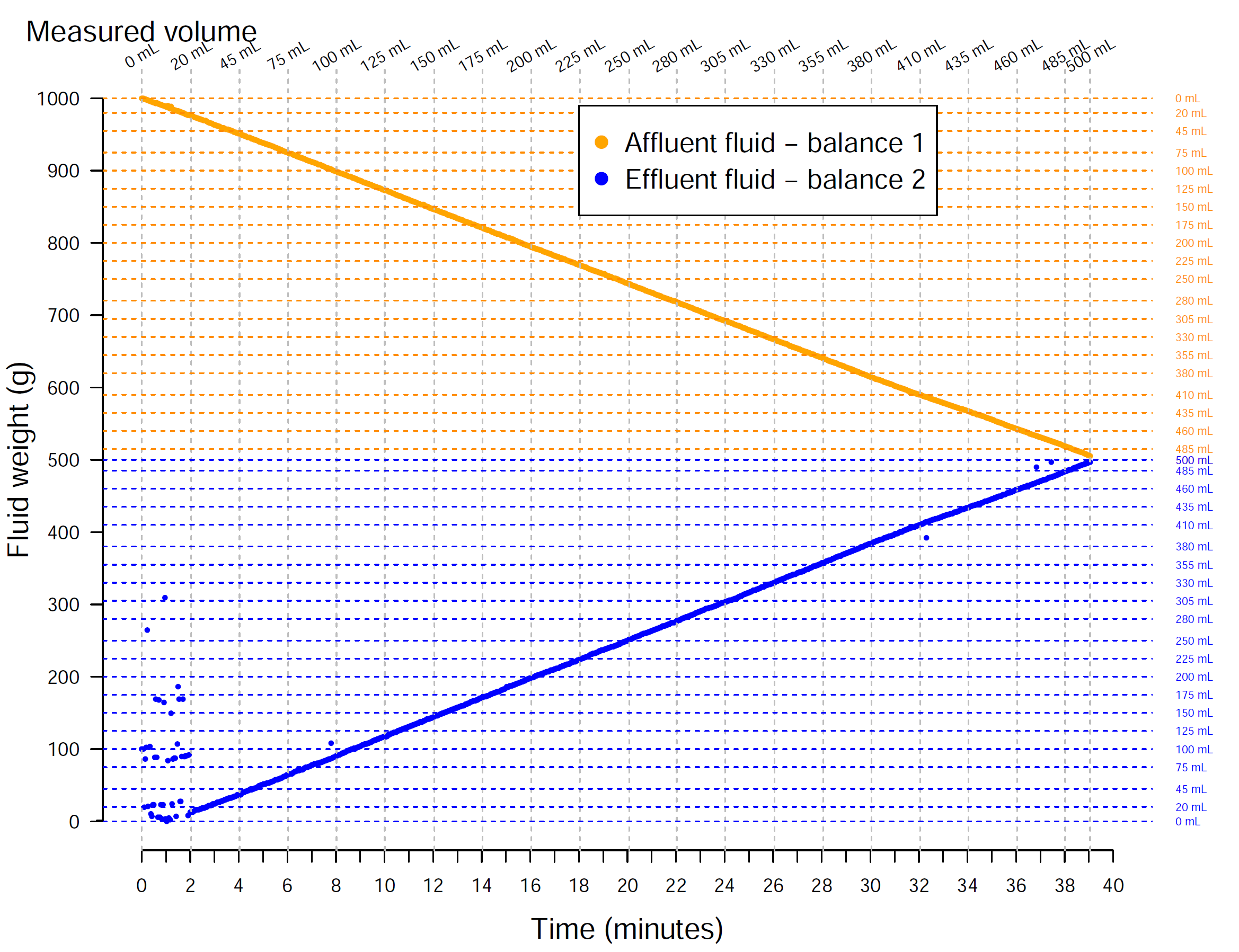


Figure S16: Pump2 working continuously with 25 bits of output from the microcontroller and transferring 500 mL of normal saline from balance 1 to balance 2.

We collected 885 timepoints values of fluid weight from each balance.

We collected the real normal saline volume transferred from balance 1 to balance 2 each approximately 2 minutes (Presented as measured volume).

The time spent to complete the fluid transfer was 39.05 minutes.


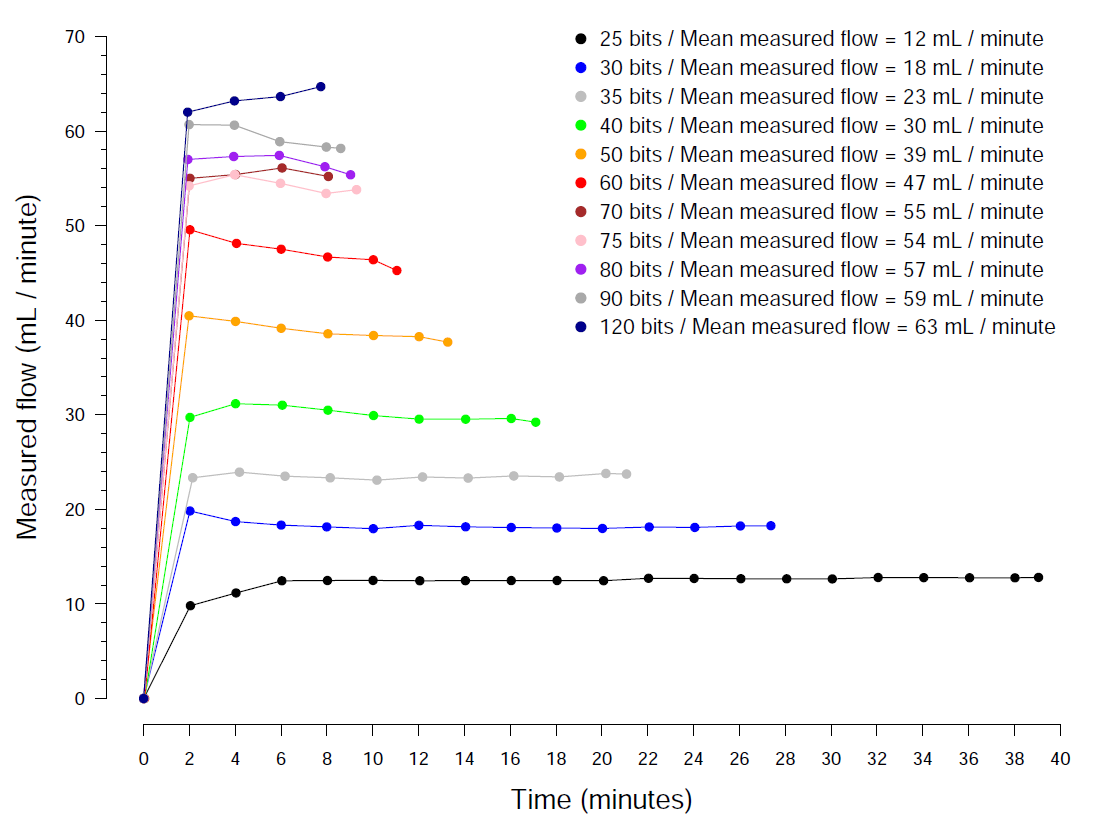


Figure S17: Fluid flow calculated trough the beaker measured volume beyond the time.

Legend shows the bits outflow from the microcontroller.

The higher the bits outflow, the higher the measured fluid flow.


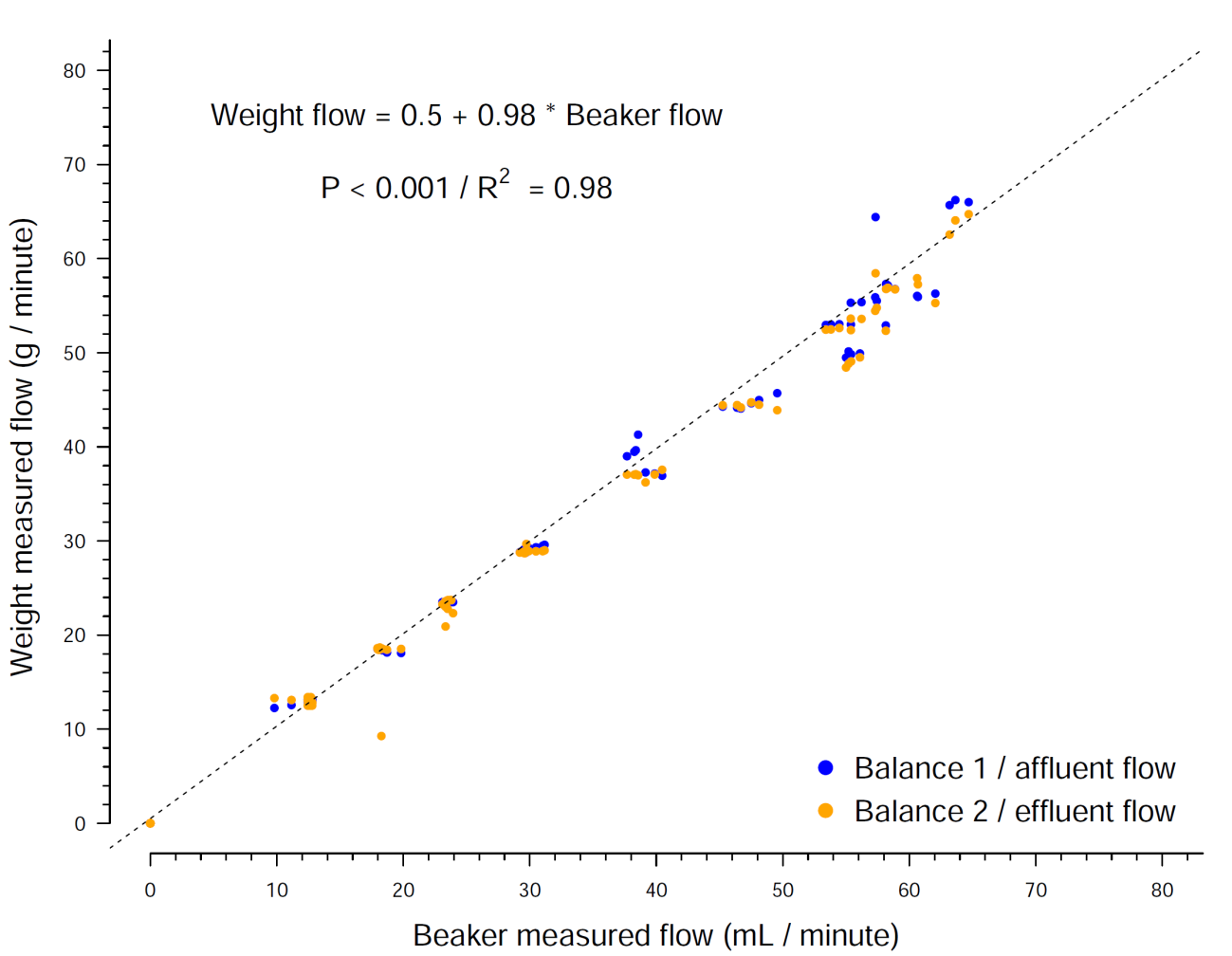


## Figure S18: Correlation of beaker measured flow and weight measured flow retrieved from both balances (affluent and effluent fluids).

A total of 202 data points were collected.


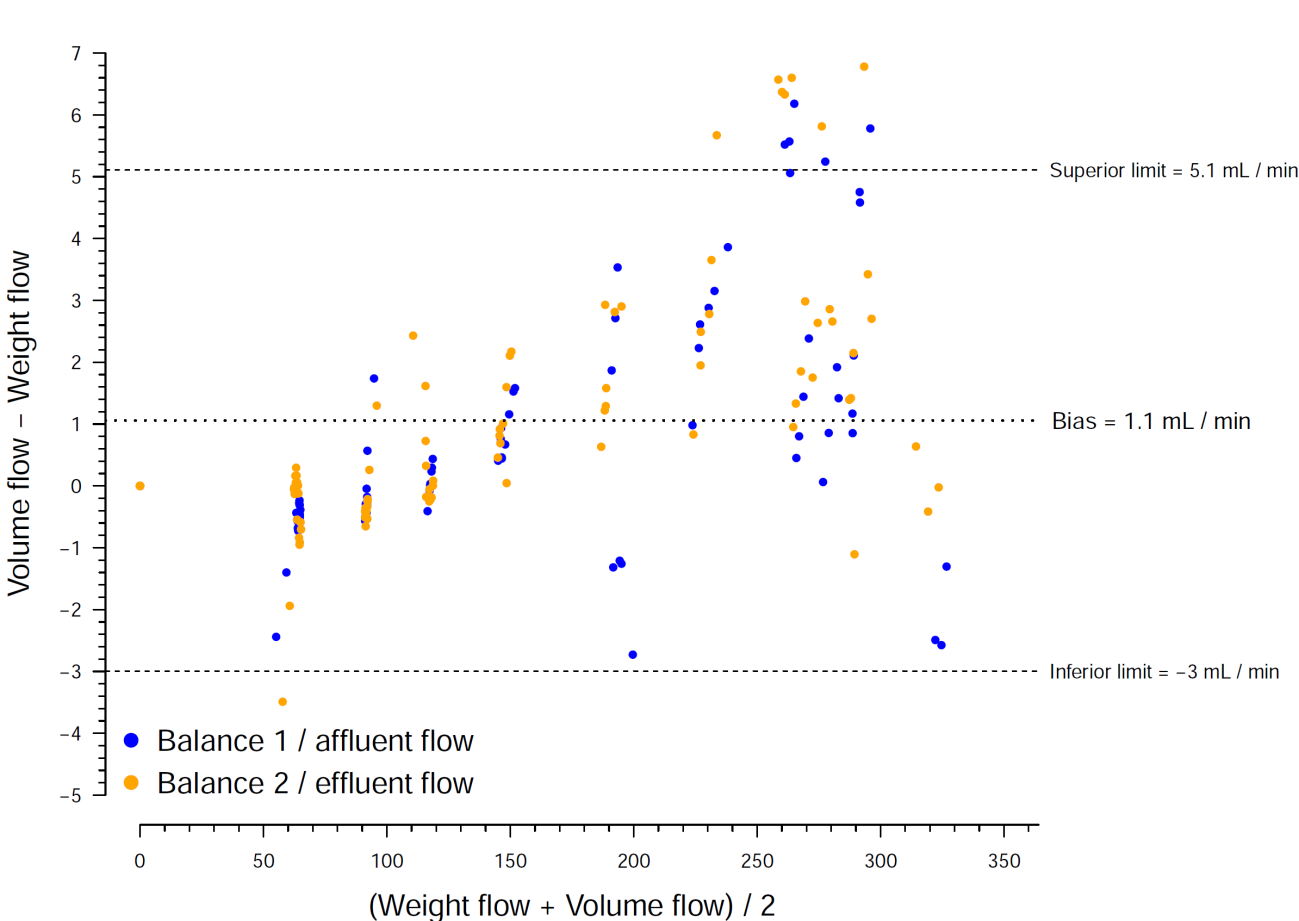


## Figure S19: Bland – Altman diagramagreement between beaker measured flow (volume) and weight measured flow using both balances (affluent and effluent).

A total of 202 data points were collected.

## Figure S20: Pump2 working intermittently (approximately 4 seconds on and 4 seconds off)with 10 bits of output from the microcontroller and transferring 500 mL of normal saline from balance 1 to balance 2.

We collected 1437 timepoints values of fluid weight from each balance.

We collected the real normal saline volume transferred from balance 1 to balance 2 each approximately 2 minutes (Presented as measured volume).

The time spent to complete the fluid transference was 63.68 minutes.

## Figure S21: Pump2 working intermittently (approximately 4 seconds on and 4 seconds off)with 5 bits of output from the microcontroller and transferring 500 mL of normal saline from balance 1 to balance 2.

We collected 1392 timepoints values of fluid weight from each balance.

We collected the real normal saline volume transferred from balance 1 to balance 2 each approximately 2 minutes (Presented as measured volume).

The time spent to complete the fluid transference was 62.3 minutes.

Figure S22: Fluid flow calculated trough the beaker measured volume beyond the time, using intermittent flow.

Legend shows the bits outflow from the microcontroller. The higher the bits outflow, the higher the measured fluid flow.


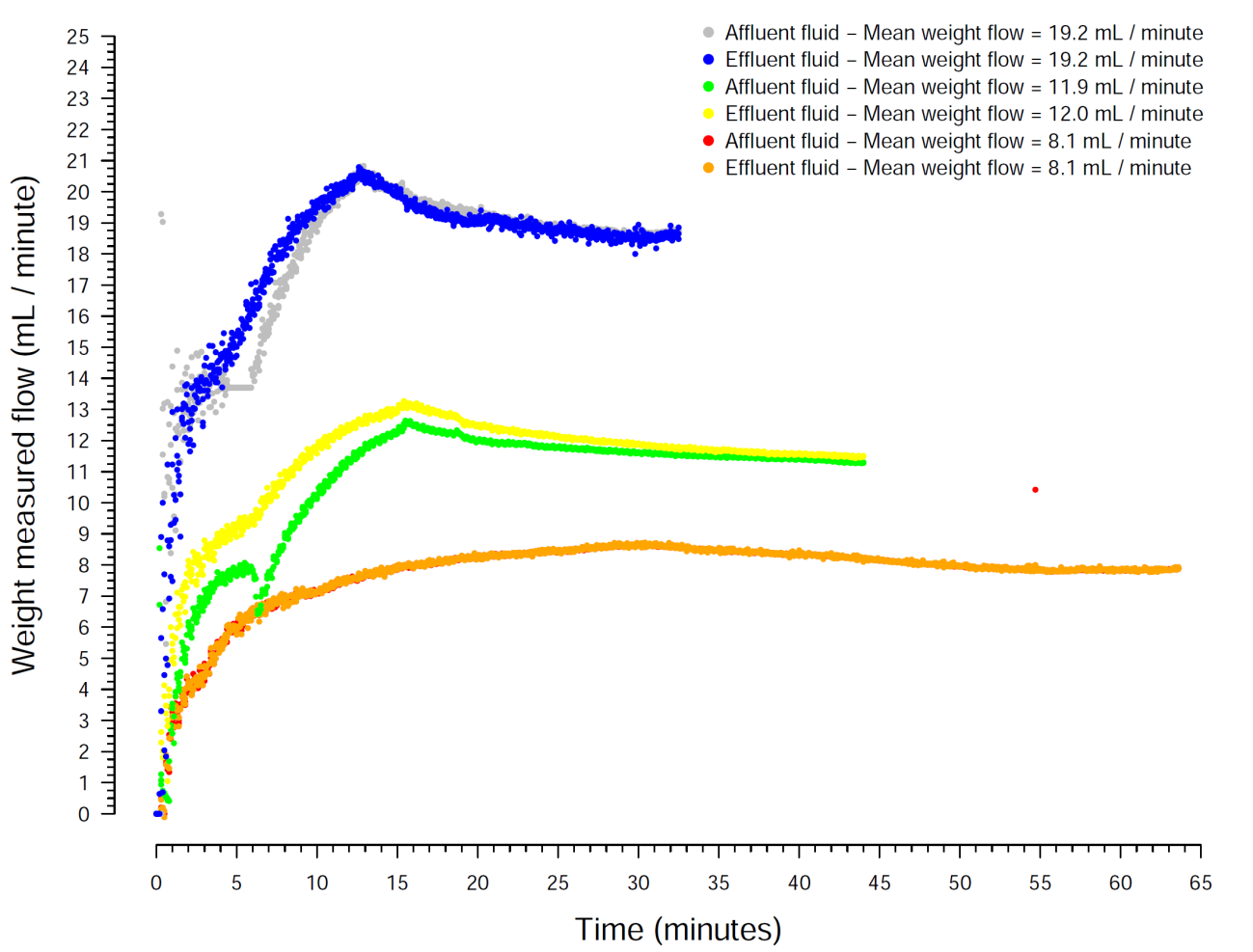


## Figure S23: Fluid flow measured through the fluid weight beyond the time, using variable intermittent value of bits and flow.

Legend shows the mean flow calculated using the fluid bags weight.

In theseexperiments, the pump 1 was used.

The fluid flow was set to 20, 12 and 8 mL/minute to each pair of curves (affluent and effluent bags) from upside to downside respectively.

The number of data points collected was 900 to the flow of 20 mL/minute, 981 to the flow of 12 mL/minute, and 1437 to the flow of 8 mL/minute.

The mean weights flow showed in the legend are the mean weight flow after 15 minutes of data collection.


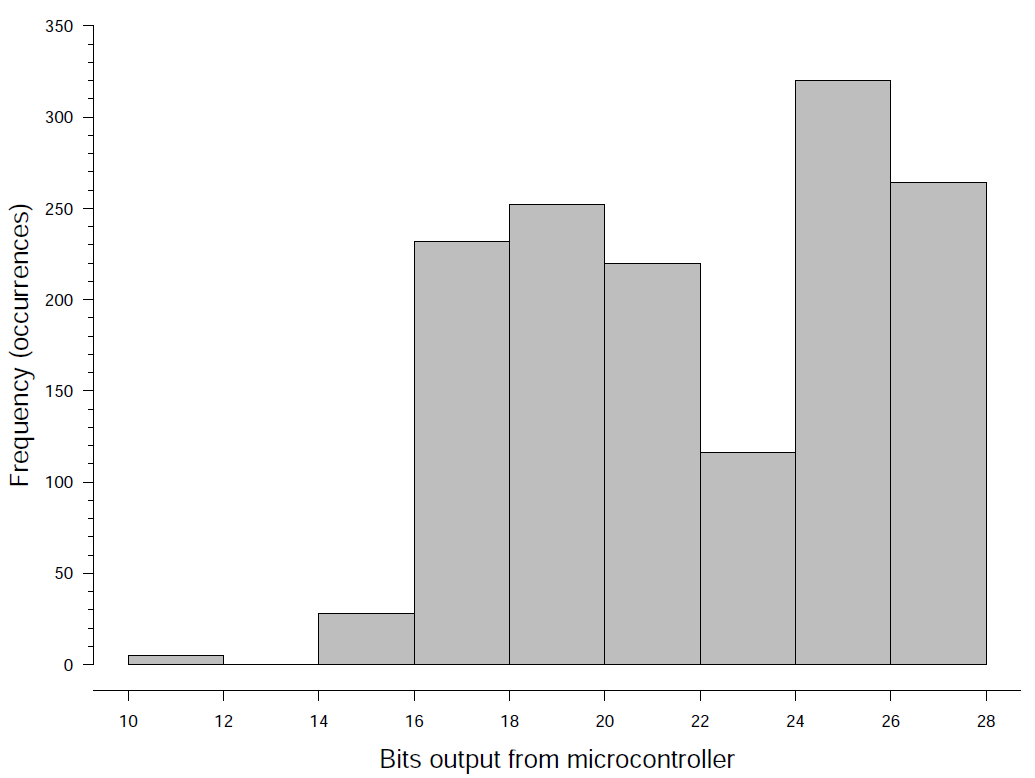


## Figure S24: Histogram showing the bits output from the microcontroller when the experiment was set to intermittent flow of 8 mL/minute.

There were 1437 data points collected.


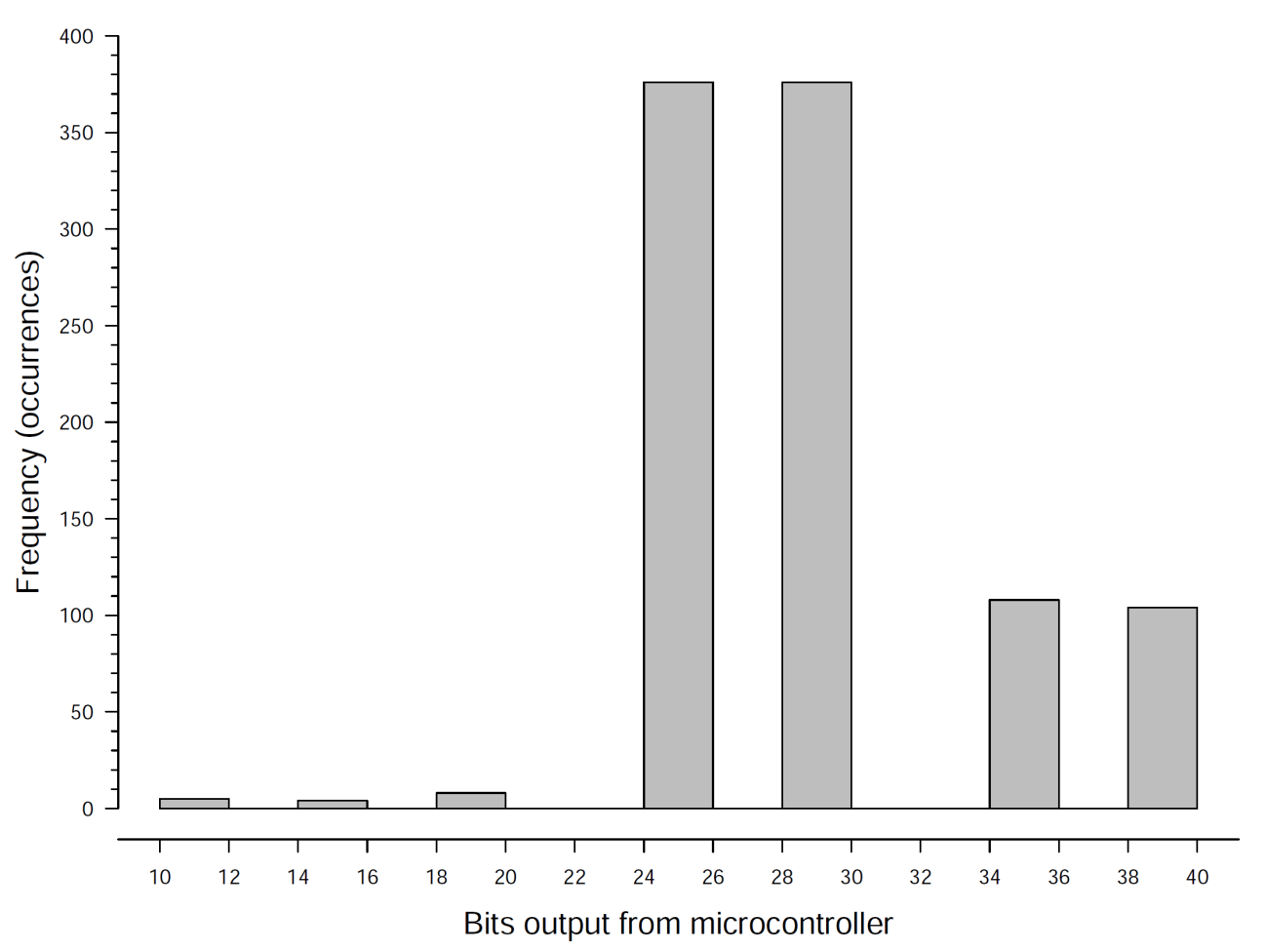


## Figure S25: Histogram showing the bits output from the microcontroller when the experiment was set to intermittent flow of 12 mL/minute.

There were 981 data points collected.


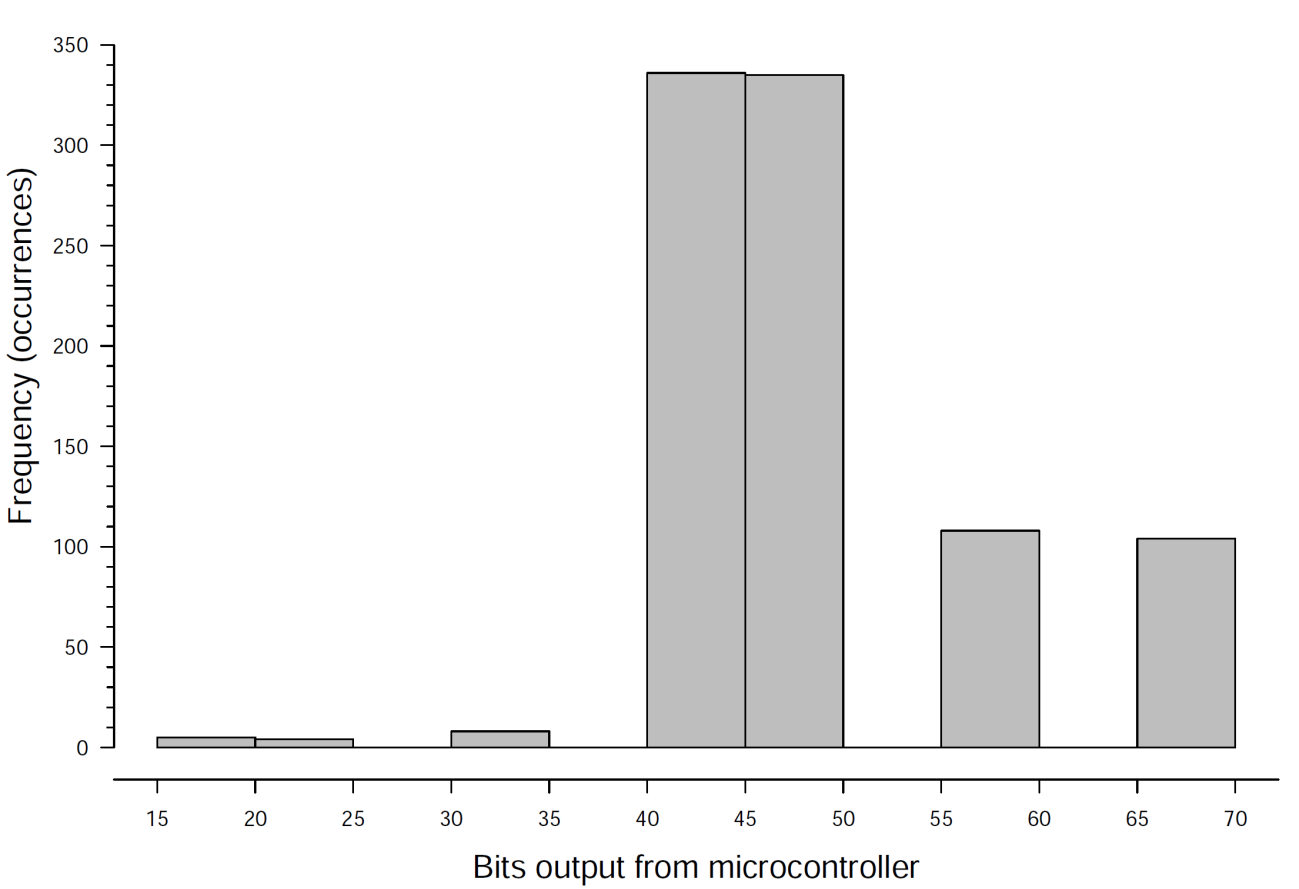


## Figure S26: Histogram showing the bits output from the microcontroller when the experiment was set to intermittent flow of 20 mL/minute.

There were 900 data points collected.


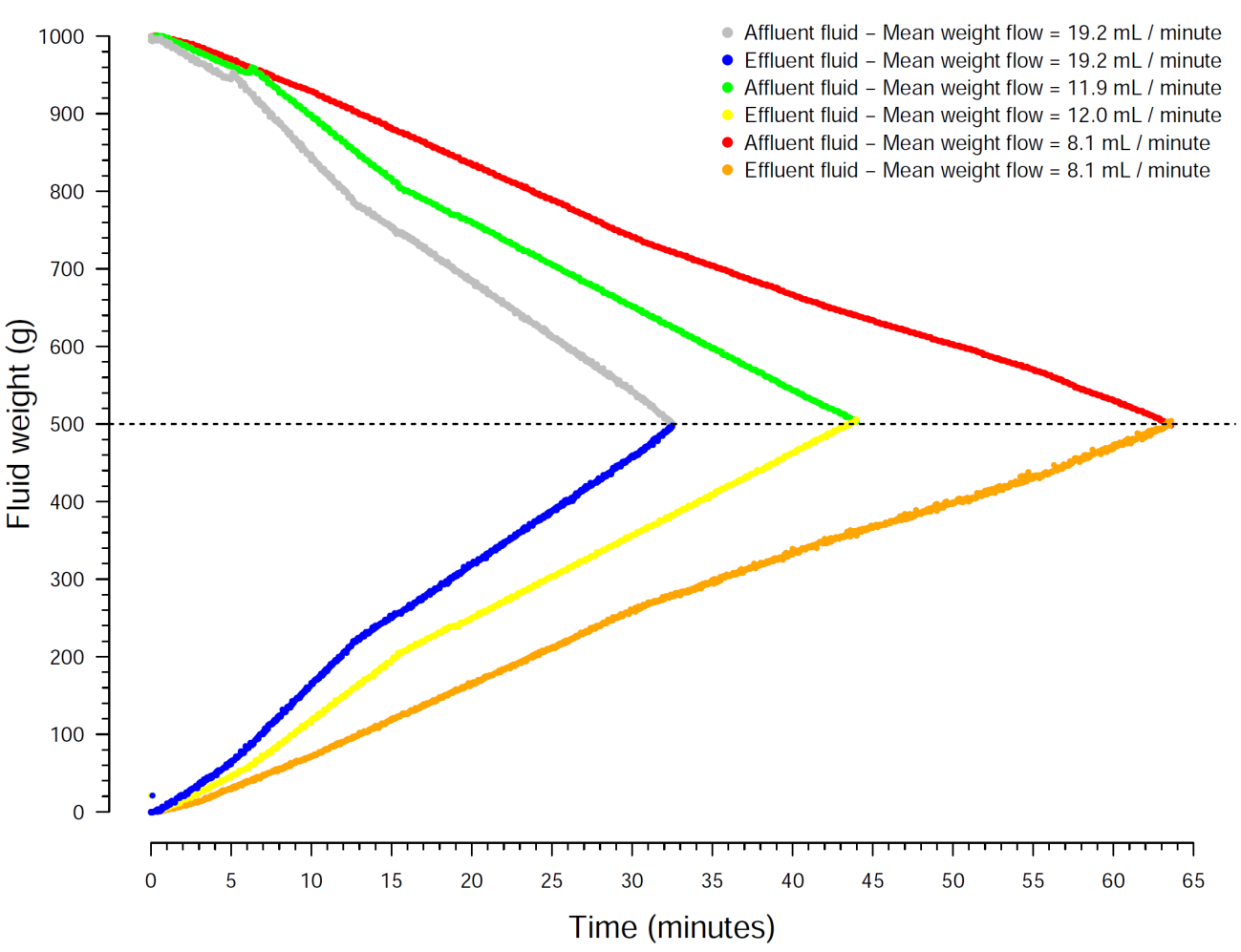


## Figure S27: Pump1 working intermittently (approximately 4 seconds on and 4 seconds off) with variable bits of output from the microcontroller and transferring a preset flow of 20 (gray and blue curves), 12 (green and yellow curves) and 8 (red and orange curves) mL/minute of normal saline from balance 1 to balance 2.

We collected 1437 (flow = 8 mL/minute), 981 (flow = 12 mL/minute) and 900 (flow = 20 mL/minute) timepoints values of fluid weight from each balance.

## Figure S28: Boxplot showing the median, P25^th^, P75^th^, minimum and maximum number of bits output from microcontroller during the two hours of experiment.

UF denotes ultrafiltration.

## Figure S29: Bar plot the percentage of the time during the two hours of experiment with pumps on and off.

UF denotes ultrafiltration.

## Figure S30: Data collectedduring the first prolonged bench experiment.

The median number of bits output from microcontroller are shown in the panel A; and the percentage of the 797 minutes of the experiment with the pumps on and off are shown in the panel B.

In this experiment, an affluent volume of 900 mL/hour and an ultrafiltration rate of 80 mL/hour were set.

There were 20396 data points collected.

## Figure S31: The affluent flow over the 797 minutes observed during the first prolonged bench experiment.

In this experiment, an affluent volume of 900 mL/hour and an ultrafiltration rate of 80 mL/hour were set.

## Figure S32: Data collected during the second prolonged bench experiment.

The median number of bits output from microcontroller are shown in the panel A; and the percentage of the 362 minutes of the experiment with the pumps on and off are shown in the panel B.

In this experiment, an affluent volume of 900 mL/hour and an ultrafiltration rate of 60 mL/hour were set.

There were 9990 data points collected.

## Figure S33: The affluent flow over the 362 minutes observed during the first prolonged bench experiment.

In this experiment, an affluent volume of 900 mL/hour and an ultrafiltration rate of 60 mL/hour were set.
